# The cell biology of HIV-1 latency and rebound

**DOI:** 10.1186/s12977-024-00639-w

**Published:** 2024-04-05

**Authors:** Uri Mbonye, Jonathan Karn

**Affiliations:** Department of Molecular Biology and Microbiology, Case Western Reserve University School of Medicine, Cleveland, OH 44106 USA

**Keywords:** HIV-1 latency, HIV-1 reservoir, Latency reversal, Epigenetic silencing, Transcription elongation, T-cell receptor signaling, P-TEFb, HIV-1 Tat, 7SK snRNP

## Abstract

Transcriptionally latent forms of replication-competent proviruses, present primarily in a small subset of memory CD4^+^ T cells, pose the primary barrier to a cure for HIV-1 infection because they are the source of the viral rebound that almost inevitably follows the interruption of antiretroviral therapy. Over the last 30 years, many of the factors essential for initiating HIV-1 transcription have been identified in studies performed using transformed cell lines, such as the Jurkat T-cell model. However, as highlighted in this review, several poorly understood mechanisms still need to be elucidated, including the molecular basis for promoter-proximal pausing of the transcribing complex and the detailed mechanism of the delivery of P-TEFb from 7SK snRNP. Furthermore, the central paradox of HIV-1 transcription remains unsolved: how are the initial rounds of transcription achieved in the absence of Tat? A critical limitation of the transformed cell models is that they do not recapitulate the transitions between active effector cells and quiescent memory T cells. Therefore, investigation of the molecular mechanisms of HIV-1 latency reversal and LRA efficacy in a proper physiological context requires the utilization of primary cell models. Recent mechanistic studies of HIV-1 transcription using latently infected cells recovered from donors and ex vivo cellular models of viral latency have demonstrated that the primary blocks to HIV-1 transcription in memory CD4^+^ T cells are restrictive epigenetic features at the proviral promoter, the cytoplasmic sequestration of key transcription initiation factors such as NFAT and NF-κB, and the vanishingly low expression of the cellular transcription elongation factor P-TEFb. One of the foremost schemes to eliminate the residual reservoir is to deliberately reactivate latent HIV-1 proviruses to enable clearance of persisting latently infected cells—the “Shock and Kill” strategy. For “Shock and Kill” to become efficient, effective, non-toxic latency-reversing agents (LRAs) must be discovered. Since multiple restrictions limit viral reactivation in primary cells, understanding the T-cell signaling mechanisms that are essential for stimulating P-TEFb biogenesis, initiation factor activation, and reversing the proviral epigenetic restrictions have become a prerequisite for the development of more effective LRAs.

## Background

In untreated people with HIV (PWH), the unabated replication of HIV-1 in CD4^+^ T cells allows the virus to evade adaptive immune responses through rapid evolution, eventually leading to the profound depletion of these cells and susceptibility to opportunistic infections (AIDS) [1, 2]. Combination antiretroviral regimens (ART), first introduced in the late 1990s, are remarkably effective at blocking HIV-1 replication and preserving CD4^+^ cell populations. Despite its success at prolonging life, ART remains imperfect since it must be administered for life and does not fully ameliorate co-morbidities such as HIV-associated neurocognitive disease, cardiovascular risk, or cancer risks. Unfortunately, ART also does little to address the viral reservoir. Prolonged viral remission after treatment interruption has only been seen in very rare individuals (< 1%) [3, 4] due to the persistence of latently infected cells. Interruption of ART almost always results in a rapid (2–8 weeks) rebound of viremia and reseeding of the virus in lymphoid tissues [5, 6]. Disappointingly, although initiation of ART within days after infection can diminish the size of the replication-competent HIV-1 reservoir and significantly delay the time to viral rebound, it does not result in ART-free remission [7]. Furthermore, HIV-1 persistence in ART-treated, virally suppressed individuals results in a chronic state of immune activation that is harmful to multiple organs and can stimulate viral rebound in the absence of ART [8].

Since T-cell sources of rebounding virus pose the ultimate obstacle to a cure for HIV-1 infection, this review will focus on recent efforts to characterize the molecular mechanisms regulating HIV-1 transcription, including host-directed mechanisms that regulate the emergence of HIV-1 from latency in primary T cells. Historically, our understanding of the control of HIV-1 transcription and rebound has been based on studies in transformed cell models, such as Jurkat cells. Unfortunately, these models do not recapitulate the underlying cell biology of infected T cells, which transition from activated effector cells to quiescent memory cells. By placing HIV-1 latency in the context of the cell biology of primary T cells, we can now provide a more accurate molecular understanding of viral dynamics and persistence.

## Main text

### The effector-to-memory transition of T cells induces HIV-1 transcriptional silencing

**Fig. 1:**
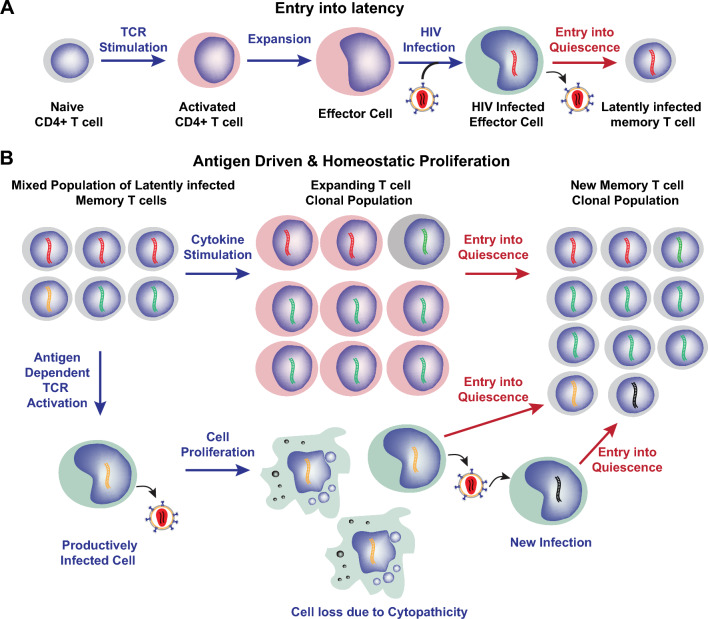
HIV reservoir formation and dynamics. **A** The reservoir is established primarily in memory CD4^+^ T cells arising during the transition of infected effector cells to achieve immunological memory. Naïve cells become activated during HIV-1 infection due to HIV-1 itself and other antigenic stimuli. The resulting activated effector cells are ideal targets for productive HIV-1 infection. A large fraction of the infected effector cells will not survive, but an important subset become quiescent and transition to a memory cell phenotype, thereby silencing HIV-1. **B** The primary mechanism for reservoir persistence is due to the clonal expansion of partially activated latently infected cells due to homeostatic proliferation driven by IL-7 or antigen stimulation. Different viral clones reactivate and expand under different conditions, with some clones being reduced or eliminated due to viral cytopathic effects. The result is a gradual simplification of the clonal population as demonstrated by recurring integration site sequences (denoted in the figure by different colors for the proviruses)

HIV-1 infects multiple CD4^+^ cell types, including macrophages [9, 10] and lymphocytes found primarily in lymph nodes [11] and gut [12]. However, the persistence of the virus during long-term ART in CD4^+^ T lymphocytes found in the blood, lymphoid organs, and mucosal lymphoid tissues requires the establishment of latency [13]. Although several groups have reported that HIV-1 latency may be a consequence of the direct infection of resting CD4^+^ T-cell subsets [14–16], particularly non-dividing follicular T helper cells in lymphoid tissues [17], overwhelming evidence, primarily derived from in vitro*-*engineered primary cell infection models, has bolstered the hypothesis that the establishment of latent HIV-1 reservoirs is a by-product of the achievement of immunological memory (reviewed in [18, 19]). Both proliferating effector T cells and effector T cells already transitioning to a resting memory state (EMT CD4^+^ T cells) may be targets of the initial infection (Fig. 1A). EMT CD4^+^ T cells express substantially higher levels of CCR5 than newly activated CD4^+^ T cells, making them more permissive to infection by CCR5-tropic HIV-1 [20]. Upon completion of reverse transcription, genome-integrated proviruses in these EMT CD4^+^ T cells are more likely to undergo a latent infection as the cells acquire a transcriptionally repressive state [20]. Since circulating memory CD4^+^ T cells, the majority of which possess a central memory phenotype, are in a quiescent state that is non-permissive for HIV-1 transcription and viral antigen production, they can evade antiviral immune surveillance.

Successful generation of latently infected primary CD4^+^ T cells ex vivo has relied on activating naïve or total CD4^+^ T cells through the T-cell receptor (TCR) as a prerequisite for permissive HIV-1 infection [21–23]. Acutely infected cells are then forced into quiescence to acquire a resting memory phenotype that establishes viral latency. Therefore, effector CD4^+^ T cells in vivo are expected to be much more permissive to HIV-1 infection since their activated state provides an intracellular environment conducive to the completion of the viral life cycle. While the majority of the productively infected cells are eliminated by viral cytopathic or host antiviral cytolytic responses, the physiological transition of a small surviving subset to quiescent memory cells substantially diminishes transcription initiation at the proviral long terminal repeat (LTR) due to the exclusion of crucial transcription initiation and elongation factors from the nucleus.

Studies showing that viral rebound is observed upon treatment interruption, even when ART is initiated within days of infection, support the idea that latency is established continuously during acute infection [24–28]. Recently, the landscape of productively infected cells at the earliest stages of HIV-1 infection was characterized by single-cell methods using the RV254/SEARCH 010 cohort which enrolls acutely infected individuals in Fiebig stages I–V [29]. Using a combination of TCR and near full-length HIV genome sequencing, they demonstrated that multiple independent infection events both in blood and lymph nodes drive the production of a relatively homogeneous viral population during acute infection and that a latent pool of cells harboring intact HIV genomes that persist during ART is established early in infection [29].

### HIV reservoir dynamics

The first evidence for persistent viral reservoirs came from studies of viral decay kinetics after ART initiation [25, 30–32]. The decay of plasma virus levels is multiphasic: During the first phase, the shutdown of viral spread combined with the short half-life (< 1 d) of T cells that produce most of the plasma virus results in a rapid and steep decline. During the second phase, viral decline slows due to the slower turnover (t_1/2_ = 14 d) of additional infected cell populations, which are often assumed to include macrophages [9]. After ~ 3 months of therapy, plasma viral loads are usually below the threshold of detection by conventional assays, but viral persistence can be monitored by looking for infected T cells carrying intact proviruses [33]. These assays show that CD4^+^ T cells with intact proviruses decay with a half-life of 19 months [34], which is still shorter than that of the latently infected cells that persist on long-term ART, estimated to have half-lives of about 44 months [35, 36].

The extended half-life of the viral reservoir seen during long-term ART is a consequence of complex cellular dynamics regulating the host T cells, resulting in a pseudo-steady state (Fig. 1B). Studies of proviral integration sites demonstrate that the HIV reservoir is maintained mainly through clonal expansion of memory T cells [37, 38]. This cellular proliferation is counter-balanced by persistent low-level rates of viral reactivation and ensuing cell death [37–40].

At the cellular level, T-cell expansion is driven by either antigen-mediated activation of T cells, which induces a waxing and waning of the infected cellular clones [40–42], or homeostatic proliferation [39] driven by cytokines such as IL-7, the central regulator of homeostatic T-cell proliferation in HIV infection [43, 44]. Ex vivo modeling has confirmed that although IL-2 and IL-7 can induce homeostatic proliferation, they do not reactivate latent proviruses [45]. Since the clonal expansion of CD4^+^ T cells does not induce viral production or enhance the clearance of the infected cell [46], it must be the sequential waves of antigen-induced expansions and contractions that result in the dynamic changes within the reservoir during ART [47].

### Molecular measurements of viral clonal expansion

The vast majority of integrated proviral HIV-1 sampled from the periphery and tissues of ART-treated virally suppressed individuals (~ 90–95%) cannot be a source of viral rebound since they are genetically defective [48–50]. While some of these defective proviruses can still be transcriptionally activated, leading to the expression of viral antigens with the potential to elicit cytolytic immune responses crucial to the clearance of infected cells [51–54], they can also have the undesirable effect of contributing to HIV-1-associated inflammation [55, 56].

The dominance of defective proviruses has significantly hampered the accurate assessment of the rebound-competent reservoir's heterogeneity and size. Developed two and a half decades ago, the quantitative viral outgrowth assay (Q-VOA) had long been considered to be a reliable measure of the proportion of circulating latently infected cells bearing inducible, replication-competent proviruses, estimated to be present at ~ 1/10^6^ CD4^+^ T cells in virally suppressed individuals [25, 27, 57]. With the realization that the reactivating stimuli used in Q-VOA can only reproducibly induce a small fraction (~ 5%) of the replication-competent reservoir in a single round [48, 58–60], it became essential to identify assays that can accurately measure the potentially inducible, subset of genetically intact proviruses in clinical and retrospective longitudinal studies.

The intact proviral DNA assay (IPDA), quadruplex quantitative PCR (Q4PCR), and multiplexed droplet digital PCR are simple PCR methods that are designed to distinguish between intact and defective proviruses. [33, 61, 62]. IPDA is a droplet digital PCR-based assay that relies on the simultaneous amplification of two subgenomic regions (packaging signal and Rev response element) to measure the proportion of intact HIV-1 proviruses in patient samples regardless of whether they are inducible, latent, or transcriptionally active [33]. Since IPDA samples a tiny fraction (2%) of the HIV-1 genome, it may incorrectly categorize a significant fraction of proviruses as intact, leading to overestimating the intact proviral reservoir. IPDA comparison of frequencies of CD4^+^ T cells with intact proviruses in matched peripheral blood (PB) and lymph node (LN) samples of ART-treated individuals surprisingly reveals a slight difference in reservoir size between the two compartments (~ 60/10^6^ vs. ~ 100/10^6^ CD4^+^ T cells in PB and LN, respectively) [50, 58]. By contrast, Q4PCR combines a multiplex qPCR for the simultaneous amplification of conserved sequences in four regions (packaging signal, *gag*, *pol,* and *env*) with a final near full-length sequencing step to improve the accuracy and sensitivity of detecting intact proviral DNA in diverse HIV-1 sequences [61]. IPDA and Q4PCR are high throughput assays that are relatively rapid, scalable, and much less labor intensive than Q-VOA or next-generation sequencing for analyzing large numbers of clinical samples. They have also proven helpful in examining long-term ART's impact on the temporal dynamics of the HIV-1 reservoir in longitudinally obtained patient samples from different cohorts.

Regardless of the assay employed, longitudinal studies of the reservoir have reproducibly demonstrated that while the defective proviral DNA is relatively stable with minimal decay observed over ten years, intact proviral sequences decay much more rapidly with an average estimated half-life of ~ 4.0 to 4.9 years [34, 63–65]. This distinctive decay pattern of the intact proviral reservoir reflects the selective pressure imparted by stochastic reactivation mechanisms on cells harboring intact proviruses, resulting in their preferential loss through viral cytopathic effects and antiviral immunity. Despite its relative instability, the reservoir of intact proviruses remains persistent with diminishing complexity over time due to its progressive enrichment in expanded clones of CD4^+^ T cells [63, 66]. Even with decades of ART adherence, the clonal expansion of cells with intact proviruses will counteract their decay, thereby increasing the reservoir with an estimated doubling time of 23 years [67].

A limitation of the IPDA and related assays is that they do not provide direct measurements of proviral inducibility. To address this problem, a variety of molecular approaches have been developed aimed at measuring the proportion of patient-derived cells that harbor transcriptionally or translationally competent HIV-1 DNA following ex vivo induction [68–73]. These include the ultrasensitive p24 assay, HIV-1-Flow, HIV-1-FISH-Flow, Tat/Rev-induced limited dilution assay (TILDA), and envelope detection of in vitro transcription sequencing (EDITS). Although these assays are limited by the inefficiency of proviral reactivation ex vivo and cytotoxic effects, like the proviral DNA assays, they allow for strong enrichment of replication-competent genomes. For example, sequence analysis of a library of HIV genomes obtained from patients has shown that EDITS, which measures the production of multiply spliced envelope mRNA in patient-derived cells, can provide a 97% enrichment of viral RNA originating from full-length HIV-1 genomes [74]. Analogously to the IPDA, this requires pairs of primers located before the major 5ʹ splice donor and within the *env* gene that are widely dispersed along the genome as well as the expression of Tat and Rev. The estimates of the size of the inducible intact proviral reservoir (~ 10–60 HIV-1 RNA^+^ cells/10^6^ CD4^+^ T cells) by EDITS corresponds closely to the estimates obtained by the IPDA assay [73, 74].

These methods also permit the phenotypic analysis of inducible HIV genomes persisting in latently infected cells. For example, a combination of single-cell sorting of p24+ cells (HIV-Flow) followed by near full-length HIV-1 genome amplification showed that there was a significantly higher proportion of clonally expanded genomes in p24+ cells compared to the pool of cells harboring non-induced and/or translation-incompetent proviruses (79% versus 50%, respectively) [75]. Remarkably, these cells were enriched for the adhesion molecule VLA-4, a combination of α4 and β1 integrins, involved in the trafficking of immune cells to inflammatory sites and in T-cell co-stimulation [75].

Since the initial studies of clonal expansion were based on total integration site analyses and were therefore biased towards the large pool of defective proviral genomes, an effort has been made to study the clonal expansion of intact proviruses. The most recent work confirms that most of the latent reservoir is maintained through clonal expansion [76–78], and that the frequency of clonally expanded cells increases over time [23]. Similarly, the most authoritative data based on single-cell, near full-length sequencing of HIV-1 DNA, coupled with proviral integration site analysis, has shown unequivocally that the total population of latently infected cells in ART-treated individuals primarily consists of heterogeneous subsets of expanded clones [37, 40, 79].

Further evidence for clonal expansion comes from analytical treatment interruption (ATI) studies investigating the genetic makeup of rebounding plasma viruses. These have highlighted that viruses derived from expanded cellular clones represent most of the rebounding reservoir [80–82]. While there appears to be little evidence for viral evolutionary clustering by anatomical site, primarily based on a comparative sequencing analysis of the variable V1–V3 *env* region of proviruses sampled from the different areas, only a tiny fraction of the rebounding viruses are genetically identical to proviruses from the cellular and anatomical sites tested during ART treatment [81]. Despite this, these ATI studies have also demonstrated no dominant anatomical reservoir correlating with HIV-1 rebound, with the rebounding virus more likely to originate from multiple CD4^+^ T-cell subsets and tissue compartments. Thus, the stochastic reactivation of latent HIV-1 that leads to viral emergence can occur in various compartments, with the genetic composition of the rebounding viruses lending support to the antigenic and homeostatic proliferation of a few infected CD4^+^ T-cell clones as being a significant driver of maintaining the rebound-competent HIV-1 reservoir.

It is noteworthy that near full-length sequencing analysis of HIV-1 DNA from PWH on long-term ART has uncovered an unequal distribution of genetically intact proviruses between the memory T-cell subsets with effector memory cells containing the most significant proportion of intact proviral HIV-1 [49]. These findings confirm the pseudo-steady nature of the memory cell HIV-1 reservoir, where the multiple states that define its equilibrium are driven by quiescence (activated effector to memory), differentiation (central to effector memory), and homeostatic proliferation. Since effector-to-memory transition and homeostatic proliferation are the major forces in creating and maintaining the latent HIV-1 reservoir, it’s been proposed that pharmacological inhibition of both processes may substantially diminish the reservoir size [66, 83].

### Residual viremia

Another potential source of viral persistence is from cells resident in anatomical sanctuary sites, such as the B-cell follicle of lymphoid tissues, genital tract, and the central nervous system that may not be adequately accessed by either ART or cytotoxic innate and CD8^+^ T cells [84–86]. However, ongoing replication during ART seems to be very rare based on molecular assays, including the presence of 2-LTR circles in infected cells [87–89], the ongoing accumulation of genetic variants [90], and the appearance of new viral integration sites [91, 92].

If the virus is not actively replicating during ART treatment, why do many PWH display persistent low-level viremia? Surprisingly, the persistent viremia may be due to a large expansion of clones of circulating infected cells in treated PWH that maintain transcriptionally active virus and survive despite host immunity through intrinsic proliferative mechanisms [51, 93–95]. Although these “repliclones” can be detected using advanced, highly sensitive single-cell and sequencing methods for detecting full-length viruses [94], they are rare compared to the reservoir of transcriptionally latent but replication-competent proviruses that are the major source of viral rebound during treatment interruption.

### Chromatin organization of proviral HIV-1 and its epigenetic silencing

As described above, the persistent forms of HIV-1 found in the reservoir are all integrated into the host cellular chromatin, which is a prerequisite to form proviral templates competent for viral RNA synthesis. During acute infection a variety of linear and circularized unintegrated DNA is also generated but these typically represent only 1 to 10% of the total viral DNA. The unintegrated HIV-1 DNA is typically compacted and transcriptionally silenced by the host epigenetic SMC5/6 complex [96–99], although a few HIV DNAs can escape this restriction and support transcription when Vpr stimulates the degradation of SMC5/6 [100]. Nonetheless, unintegrated DNA does not persist in infected T cells for more than a few days [101] and therefore does not contribute to the persistent HIV reservoir.

HIV-1 preferentially integrates into a broad but non-random range of sites in actively transcribing cellular genes [102–106]. These sites are specified by the interactions between the viral integrase, capsid and cellular cofactors [107, 108]. Binding of integrase with lens epithelium-derived growth factor (LEDGF/p75), a protein that binds to the nucleosomes of transcriptionally active genes, directs the HIV-1 integrase to transcriptionally active genes at the time of infection [109, 110]. In addition, the cleavage and polyadenylation specificity factor 6 (CPSF6), a component of the RNA cleavage and polyadenylation machinery, mediates the nuclear import of intact viral cores [111–113] and the intranuclear trafficking of viral pre-integration complexes [114, 115]. Since CPSF6-capsid interactions allow the virus to bypass peripheral heterochromatin and penetrate euchromatic regions in the nucleus, it can enhance proviral integration into transcriptionally active genes [115, 116]. HIV-1 targets super-enhancers (SEs) and speckle-associated domains (SPADs) [113, 117] within these highly transcriptionally active genomic regions.

**Fig. 2:**
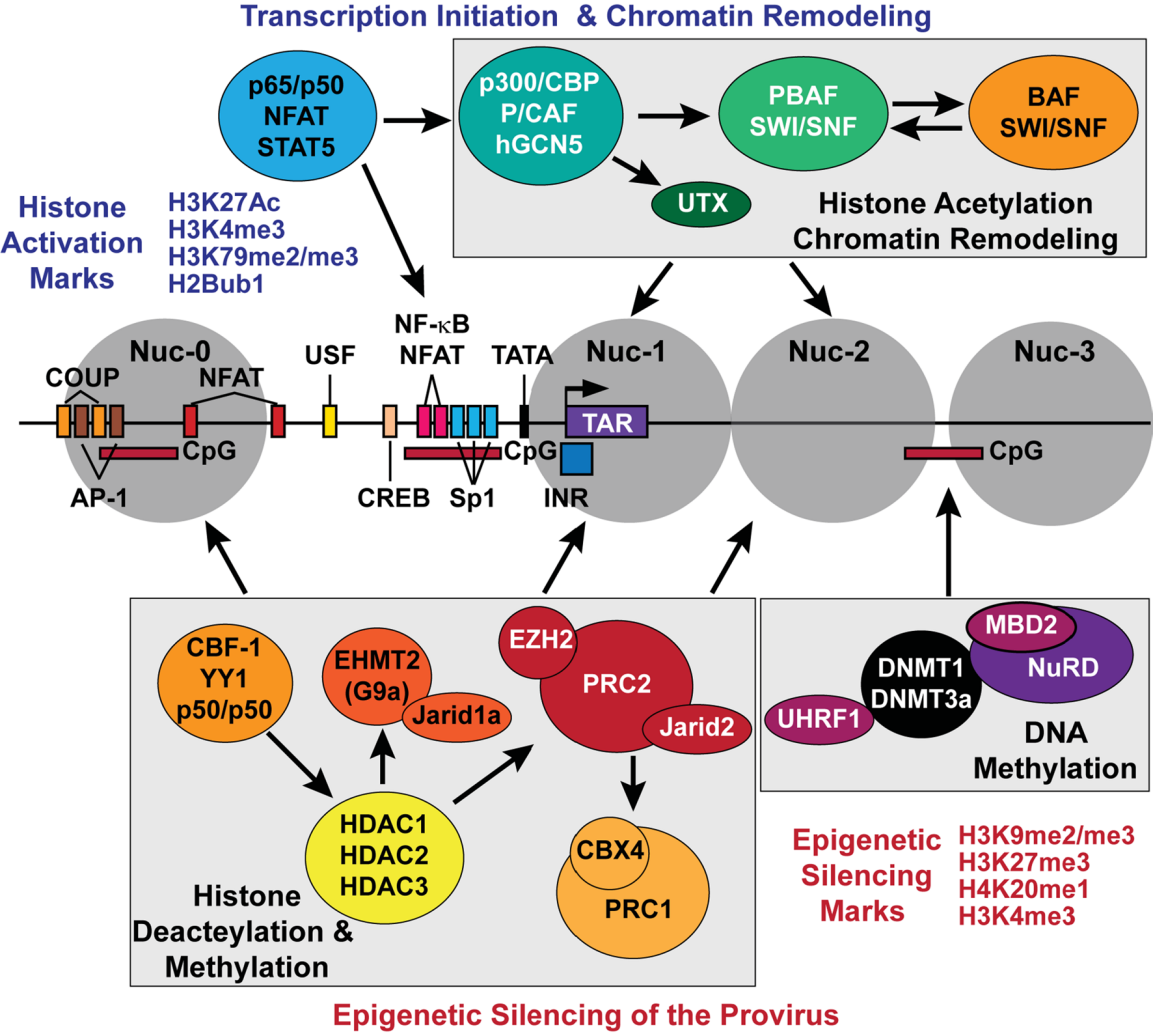
Epigenetic control of HIV-1 transcription initiation. The structure of the HIV-1 LTR and flanking nucleosomes (Nuc-0, Nuc-1, and Nuc-2) is shown at the center. In the latent state, the proviral promoter is bound by repressive trans-acting factors, including CBF-1, YY1, and NF-κB p50/p50, which direct the recruitment of histone deacetylase enzymes (HDACs). Subsequent occupancy of the promoter by the polycomb repressive complex 2 (PRC2) and EHMT2 results in the methylation of the deacetylated histone H3 at Lys27 and Lys9 positions, respectively. PRC2 also functions to recruit the polycomb repressive complex 1 (PRC1), in part via the binding of CBX4 to H3K27me3. Methylation of the 5’ CpG island (5ʹ CpGI) by DNMT1 and DNMT3a promotes the repressive histone methylation status of Nuc-1 by mediating the recruitment of UHRF1 and the HDAC-containing NuRD complex through MBD2. The SWI/SNF chromatin remodeling complex BAF interacts with Nuc-1 and is required to maintain increased Nuc-1 density around the HIV-1 transcription start site. By associating with CBX4, CAF-1, and PML, latent proviruses are likely to be situated in liquid–liquid phase-separated (LLPS) nuclear condensates that may concentrate transcriptionally poised genes with repressive heterochromatic features. CAF-1 may also play a central role in the initial assembly of nucleosomes at the provirus following integration and DNA replication. Typical of bivalent promoters, the HIV-1 LTR is in a reversible epigenetically repressed state poised for rapid inducible transcription. Recruitment of histone acetyltransferases, the H3K27me3 demethylase UTX/KMDA, and the PBAF SWI/SNF remodeling complex to the HIV-1 promoter following the nuclear induction of transcription activators enables the displacement of Nuc-1 and Nuc-2 thereby stimulating viral transcription initiation

Regardless of the chromosomal integration site, proviral HIV-1 is occupied by precisely positioned nucleosomes, Nuc-0, Nuc-1, and Nuc-2, which regulate the binding of either repressive or stimulatory host transcription factors [118] (Fig. 2). The resulting proviruses adopt an autonomous chromatin structure that eventually makes them susceptible to silencing by host epigenetic mechanisms independent of the transcriptional activity of the surrounding host genes due to insulator elements. Enhancer-blocking insulator elements are highly conserved across all eukaryotes and permit coordinated and autonomous gene expression throughout the entire genome [119].

The mechanisms that insulate HIV from the host chromosomal control remain incompletely understood but the main architectural insulator protein CCCTC-binding factor (CTCF) seems to play a central role [117, 120–122]. Although the HIV LTR does not contain CTCF binding sites, genome organization analysis reveals dynamic CTCF clusters in cells with active and repressed HIV-1 transcription. CTCF-enriched topologically associated domain (TAD) boundaries with signatures of transcriptionally active chromatin are HIV-1 integration determinants in both microglia and CD4+ T cells, and CTCF removal impairs viral integration, highlighting the importance of host genome organization in HIV-1 infection [122]. Depletion of CTCF inhibited HIV-1 latency establishment in primary CD4^+^ T cells due to its transcriptional repressor function [123]. Thus, HIV preferentially integrates into regions where it can respond to transcriptional signals independently of the surrounding host genes.

The positioning of the nucleosomes on the HIV-1 provirus is regulated during latency. In latently infected T cells, the SWI/SNF chromatin remodeling complex BAF interacts with Nuc-1 and is required to maintain increased Nuc-1 density around the HIV-1 transcription start site [124, 125]. By contrast, following T-cell activation, the closely related PBAF complex replaces BAF and facilitates the displacement of Nuc-1, thereby contributing to the activation of viral transcription [124, 126, 127]. A high throughput screen for intracellular small molecule inhibitors of BAF has led to the identification of a macrolactam capable of reversing HIV-1 latency in a primary cell model and patient-derived CD4^+^ T cells without inducing toxicity or T-cell activation [128].

The HIV-1 LTR itself is a bivalent promoter that characteristically contains both the activating histone H3 Lys4 tri-methylation (H3K4me3) and repressive H3K27me3 marks on Nuc-1 situated around the proviral transcription start site [129–131]. Thus, the LTR of latent HIV-1 is in a reversible epigenetically repressed state poised for rapid inducible transcription, as is typical for cellular bivalent promoters [132, 133]. Transcriptional silencing of HIV-1 is strongly associated with the occupation of the HIV-1 LTR by transcriptional repressors such as CBF-1, YY1, and NF-κB p50 homodimers that recruit histone deacetylase (HDAC) and methylation enzymes leading to the formation of an array of specific histone modifications at Nuc-1 [129, 134–143] (Fig. 2).

Two of the best characterized repressive heterochromatin marks on proviral HIV-1 are H3K27me2/me3 and H3K9me2/3 which are conferred by EZH2, the catalytic component of the polycomb repressive complex 2 (PRC2), euchromatic histone-lysine *N*-methyltransferase 2 (EHMT2, also known as G9a methyltransferase) and SUV39H1 (Suppressor of Variegation 3–9 Homolog 1). Various combinations of histone lysine methyltransferase enzymes occupy the HIV-1 promoter in cell line models of viral latency, but they are all rapidly displaced from the promoter upon proviral reactivation [129, 131, 144]. The relative contributions of each methyltransferase to HIV latency have been difficult to dissect because of heterogeneity in the epigenetic regulation of HIV in different Jurkat clones [130]. For example, CRISPR-based knockout of EZH2 expression in latently infected Jurkat E4 cells was found to also cause the depletion of EHMT2, leading to a modest proviral reactivation or potentiation of latency reversal by the HDAC inhibitor SAHA [129]. By contrast, CRISPR-mediated knockout of EHMT2 was selective for EHMT2 and failed to produce any inducible effects on latent proviruses in this Jurkat model. Similarly, although SUV39H1 occupies the HIV-1 promoter in tandem with the H3K9me3 reader HP1γ and an HDAC1/2 complex [140, 144], its depletion by RNA interference, or inhibition of its H3K9me2/3 activity with chaetocin, did not effectively reactivate latent HIV-1 proviruses in the Jurkat E4 model [129, 131], suggesting that the methylation of H3K9 by either SUV39H1 or EHMT2 may be insufficient to reverse proviral latency in this model. By contrast, in several J-Lat cell clones, partial viral reactivation by chaetocin treatment or SUV39H1 shRNA knockdown was observed [145–147]. Inhibitor-based studies of the lysine methyltransferase activity of EZH2 (by GSK-343 or EPZ-6438), EHMT2 (by UNC-0638) or SUV39H1 (by chaetocin) in resting CD4^+^ T cells isolated from virally suppressed PWH or in a Th17 primary cell model of HIV-1 latency have suggested that each of these enzymes contribute to establishing and maintaining proviral transcriptional silencing in primary CD4^+^ T cells [129, 135, 148]. Given the multiple restrictions on HIV transcription in resting memory cells, it is not surprising that reversing epigenetic silencing by histone methyltransferase inhibition typically results in only modest reactivation, but methyltransferase inhibitors are often synergistic with complementary latency-reversing agents [21]. Mono-methylation of histone H4 at lysine 20 (H4K20me1) by SMYD2 has also been shown to contribute to establishing HIV-1 latency in primary T cells [147].

As illustrated in Table 1, distinct epigenetic mechanisms are known to regulate the transcription of proviral HIV-1 in primary infected T cells [129, 149] and brain microglia [122]. In both cell types, viral latency appears to be reinforced primarily by creating repressive heterochromatin structures at the provirus that may influence the intranuclear reorganization of the proviral genome into phase-separated condensates that tightly regulate local transcription. For instance, in infected CD4^+^ T cells, the enrichment of chromatin assembly factor 1 (CAF-1) at the LTR of latent proviruses enables this histone chaperone to mediate the formation of nuclear bodies with liquid–liquid phase separation properties that are rich in epigenetic modifiers and histone remodeling enzymes essential for establishing and maintaining HIV-1 latency [150] (Fig. 2). CAF-1 may also have a central role in the initial assembly of nucleosomes at the provirus following integration and DNA replication [151, 152]. Similarly, CBX4, a component of the Polycomb Repressive Complex 1 (PRC1), can mediate the formation of phase-separated nuclear bodies while also facilitating the recruitment of the EZH2 subunit of PRC2 to the HIV-1 LTR [153]. Consequently, CBX4 is thought to act as a bridge between the repressor complexes PRC1 and PRC2 to coordinate the maintenance of HIV-1 latency. CBX4 also SUMOylates EZH2, thereby enhancing H3K27 methyltransferase activity of EZH2 [153]. Transcriptionally silenced HIV-1 proviruses have also been shown to reside near promyelocytic leukemia (PML) nuclear bodies where the binding of PML to the latent HIV-1 promoter coincides with the presence of repressive heterochromatic marks, most notably the EHMT2-induced H3K9me2 [154]. Thus, the disruption of CAF-1, CBX4, and PML phase-separated nuclear condensates that concentrate latent proviruses with repressive heterochromatic features has been proposed to be a potential strategy for reversing HIV-1 latency in CD4^+^ T cells [150, 153–155].

**Table 1:** Epigenetic factors known to have a repressive or activator role in regulating HIV-1 transcription in T cells and/or microglia

Epigenetic factor		Identified HIV-1 transcriptional regulatory function by cell type				
Repressive epigenetic enzymes	Histone or DNA modification	Primary T cells	Transformed T cells	Primary microglia	Transformed microglia	Selected references
HDAC1, 2, and 3	Deacetylation of multiple H3 and H4 sites	+	+	+	+	[135, 139, 416–418]
EZH2 (PRC2)	H3K27me3	+	+	+	+	[129, 135, 419]
EHMT2 (G9a)	H3K9me2	+	+	+	+	[129, 419]
SUV39H1	H3K9me3	+	+	?	+	[129, 144, 148, 420]
SMYD2	H4K20me1	+	+	?	?	[147]
DNMT1; DNMT3a	meCpG	+	+	?	?	[130, 157, 163]
LSD1		?	+	?	+	[304, 421]
KAT5 (Tip60)	H4K5Ac, H4K8Ac, H4K12Ac	+	+	?	?	[315]

Activating epigenetic enzymes	Histone or Tat modification					
CBP/p300	H3K27Ac and other H3 and H4 acetylation sites; Tat K50Ac and K51Ac	+	+	+?	+?	[134, 135, 418, 422, 423]
GCN5	Primarily H3K14Ac; Tat K50Ac and K51Ac	+?	+?	+?	+?	[290, 418, 423]
PCAF	H3K9Ac, H3K14Ac; H4K8Ac; Tat K28Ac	+?	+?	+?	+?	[291, 418, 423]
UTX/KMD6A	Demethylation of H3K27me3	+	+	?	?	[130]

Epigenetic mediators	Associated partners or complex					
Nurr1	CoREST, HDAC1, G9a, EZH2	−	−	+	+	[419]
CTIP2	LSD1, HIC1, HMGA1, HDAC1, HDAC2, SUV39H1, HP1-γ	−	−	?	+	[140, 420, 421, 424]
MBD2	NuRD	+	+	?	?	[157]
SWI/SNF	BAF and PBAF	+	+	?	?	[124–128]
CHAF1a	CAF1	+	+	?	?	[150]
CBX4	PRC1	+	+	?	?	[153]
PML	PML-G9a	+	+	?	?	[154]

(+) indicates evidence of HIV-1 regulatory function by cell type. For the histone acetyltransferases, (+) is a direct demonstration of function based on ChIP assays showing recruitment to the HIV-1 LTR. By contrast, (+?) indicates indirect evidence of possible histone acetyltransferase function based on experimental work conducted using HDAC inhibitors. (−) indicates evidence that the factor either does not participate in or has a non-essential HIV-1 regulatory function in that cell type. (?) indicates that HIV-1 regulatory function is yet to be demonstrated in that cell type

In addition to histone methylation, hypermethylation of proviral DNA mainly detected at two CpG islands flanking the transcription start site has been associated with HIV-1 silencing in both latently infected Jurkat T cells and primary CD4^+^ T cells [156, 157]. Proviral DNA methylation by DNMT1 and DNMT3a is thought to mediate the recruitment of the NuRD complex via the methyl-CpG binding domain protein MBD2 [157]. However, these findings are inconsistent with observations made in latently infected CD4^+^ T cells from ART-treated virally suppressed PWH where proviral DNA methylation was very low or absent [158].

The ability of epigenetic factors to silence HIV-1, suggested that it might be possible to exploit these mechanisms to permanently silence HIV-1—the “Block and Lock” strategy for an HIV cure [159]. In a primary T cell model of HIV-1 latency, ATACseq studies have shown that chromatin accessibility of the proviral genome is significantly reduced when the provirus becomes latent, signifying the acquisition of physical and epigenetic barriers to viral gene expression [123]. Interestingly, while the genomic site of proviral integration influences the response to latency-reversing agents [160], integration sites of intact proviruses from elite controllers are observed to be located further away from the accessible chromatin and tend to be more enriched in repressive chromatin marks [161]. These observations have suggested that the heterochromatic nature of proviral reservoirs in elite controllers may also contribute to their ability to control viral replication spontaneously [161].

There have been some promising reports that an analog of cortistatin A, dCA, can not only block HIV Tat activity but also induce epigenetic modifications that lead to long-term silencing of HIV proviruses [162]. However, an attempt to permanently silence latent HIV-1 in infected primary Th17 cells by pharmacological inhibition of the prominent cellular H3K27me3 demethylase, UTX/KMDA, was unsuccessful [130]. Although UTX/KMDA inhibition or knockdown led to an enhancement in H3K27me3 levels and stimulated CpG DNA methylation by DNMT1 on both the proviral LTR and its gene coding region in ex vivo infected primary T cells and suppressed the reactivation of latent HIV-1 in memory CD4^+^ T cells isolated from ART-treated PWH, withdrawal of the UTX inhibitor also led to a rapid DNA-demethylation of the HIV-1 LTR accompanied by a reversal of transcriptional suppression [130]. CpG DNA methylation at the HIV-1 promoter is also confirmed to promote the repressive histone methylation status of Nuc-1 by facilitating the recruitment of the integrator factor UHRF1, which coordinates the chromatin assembly of DNMT1 and G9a/EHMT2 enzyme complexes [163]. Thus, the induction of restrictive epigenetic structures through cooperative histone and DNA methylation processes is necessary for HIV silencing but appears insufficient to permanently block HIV-1 proviral transcription.

### Initiation of HIV transcription

The emergence of HIV-1 from latency requires the transactivation of epigenetically silenced proviruses by host transcription factors (Table 2) and the viral factor Tat, which delivers the host transcription elongation factor P-TEFb. Despite the specialized transactivation mechanism involving Tat, the HIV-1 promoter possesses a complement of *cis*-acting elements found in many cellular promoters. The viral core promoter of HIV-1 subtype B is highly conserved genetically [164] and comprises three tandem Sp1 binding sites, a TATA box, and an initiator element at the transcription start site (Fig. 2). The binding of TFIID to core promoter DNA nucleates the recruitment of the rest of the transcription preinitiation complex and occurs independently of Tat [165].

**Table 2:** Transcription factors or events known to regulate HIV-1 transcription in T cells and/or microglia

Transcription factor or stage	Identified HIV-1 transriptional regulatory function by cell type				
DNA-binding repressors	Primary T cells	Transformed T cells	Primary microglia	Transformed microglia	Selected references
CBF-1	+	+	?	−	[135]
ESR1	+	+	?	−	[72]
NF-κB p50/p50	?	+	?	?	[142, 425]
Sp1	?	+	?	+	[426, 427]
AP-1 (c-Fos/c-Jun)	+	−	?	?	[428, 429]
c-Myc	?	+	?	?	[427]
YY1/LSF	?	+	?	?	[138, 139, 430]
TRIM28	?	+	?	?	[330, 331]
GR	?	−	?	+	[431]
CTIP2	?	?	?	+	[138]

Activating initiation factors					
NFAT	+	−	−	−	[21, 22, 167, 174, 432]
NF-κB p65/p50	+	+	+	+	[21, 22, 167, 168, 174, 432]
NF-κB p52/p100	+	+	?	?	[390, 391]
AP-1 (c-Fos/c-Jun)	?	+	?	?	[174, 429]
Sp1	?	+	?	+	[433, 434]
STAT5	+	?	?	?	[187]
IRF3	−	−	+	+	[435]
RBF-2	?	+	?	?	[169, 324]
TRIM28 (KAP1)	?	+	?	?	[325]
COUP-TF	−	−	?	+	[433, 436]
NF-IL6 (C/EBPβ)	−	+	?	+	[426, 437]

Transcription elongation					
Promoter-proximal pausing	+	+	?	?	[262, 313]
P-TEFb requirement	+	+	?	+	[262, 371, 438]
Cyclin T1 expression	Inducible	Constitutive	?	Constitutive	[364, 365, 371, 438]
CDK9 expression	Constitutive	Constitutive	?	Constitutive	[364, 365, 371, 438]
7SK snRNP assembly	Inducible	Constitutive	?	Constitutive	[240, 262, 364, 438]
SEC requirement	?	+	?	?	[439, 440]
ELL2 expression	Inducible	Inducible	?	?	[240, 440]

P-TEFb or SEC recruitment factors					
HIV Tat requirement	+	+	?	+	[214–216, 441]
TRIM28	+	+	?	+	[313, 325, 442]
HSF-1	+	+	?	?	[323, 328]
HMGA1	?	?	?	+	[438]
BRD4	+	+	?	?	[272, 315, 316]

(+) indicates evidence of HIV-1 regulatory function by cell type. (−) indicates evidence that the factor either does not participate in or has a non-essential HIV-1 regulatory function in that cell type. (?) indicates that the HIV-1 regulatory function is yet to be demonstrated in that cell type. Also shown is a classification by cell type of the known or unknown expression profiles of P-TEFb subunits, 7SK snRNP and the SEC component ELL2

Latent HIV-1 proviruses constitutively carry the Sp1 transcription initiation factor but cannot recruit RNA polymerase II (RNAP II) because of the epigenetic restrictions imposed on the provirus [22, 166]. Antigen stimulation of memory T cells activates the TCR and initiates a cascade of signaling pathways leading to the rapid nuclear induction and assembly of host transcription initiation factors such as NFAT, NF-κB, AP-1, STAT5, and the Ras-responsive binding factor-2 (RBF-2). These factors bind directly to well-defined cognate *cis*-acting elements at the proviral promoter [167–175] (Fig. 2).

The duplicated *cis*-acting NF-κB elements at the proviral HIV-1 promoter overlap with the elements recognized by NFAT, implying that these transcription initiation factors can only act in a mutually exclusive manner. The binding of NF-κB to the promoter is insufficient to induce proviral gene expression but also requires a cooperative interaction with constitutively bound Sp1 [176]. In the context of TCR signaling, inhibitor experiments in primary cell latency models have suggested that NFAT is the dominant transcription initiation factor [21, 22]. A plausible hypothesis is that NFAT and NF-κB are sequentially recruited to the proviral promoter, with the former being essential for mediating the earlier phase of transcription. Similarly, the recruitment of AP-1 to the proviral promoter is also likely enhanced by its physical interaction with NFAT or NF-κB [174, 177–180].

**Fig. 3:**
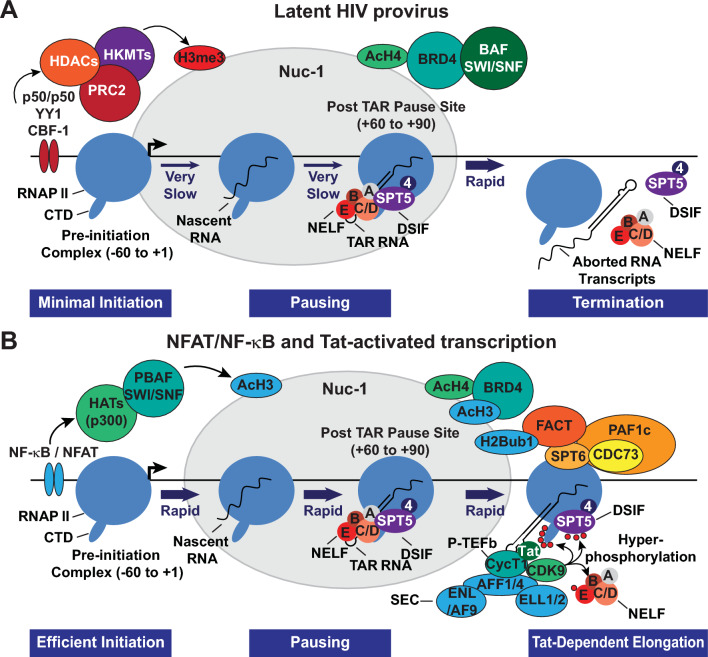
Stimulation of efficient HIV-1 transcription elongation through Tat-dependent P-TEFb recruitment and remodeling of the chromatin barrier. The current understanding of the regulation of processive HIV-1 transcription, as portrayed here, is based mainly on studies conducted using cell line models. **A** Epigenetic repressive features at the proviral promoter prevent the recruitment of RNA polymerase II (RNAP II) and assembly of the preinitiation complex, thereby restricting the expression of Tat. Latent proviruses also characteristically possess elevated acetylated histone H4 (AcH4) levels that permit their occupancy by the short isoform of BRD4, which reinforces viral latency through direct recruitment of BAF SWI/SNF complexes. Without Tat expression, an accumulation of inefficiently transcribing promoter-proximally paused RNAP II complexes due to NELF and DSIF activity may lead to abortive transcription. **B** Chromatin remodeling and efficient assembly of preinitiation complexes may initially allow for the onset of Tat-independent transcription elongation likely occurring through the recruitment of P-TEFb by NF-κB, BRD4, TRIM28, or HSF1. Synthesized Tat efficiently trans-activates HIV-1 transcription elongation by outcompeting BRD4 for P-TEFb binding and recruiting P-TEFb with the super elongation complex (SEC) to the TAR hairpin. P-TEFb eventually phosphorylates the RNAP II C-terminal domain (CTD), its linker region between the polymerase core and CTD, the SPT5 subunit of DSIF, and the NELF-E subunit. Phosphorylated DSIF is transformed into a positive elongation factor, while NELF-E phosphorylation leads to the dissociation of the NELF complex from RNAP II. The phosphorylated RNAP II CTD and linker provide a scaffold to anchor the histone chaperone SPT6, which, along with FACT, is essential in enabling the elongating machinery to transcribe through nucleosomal barriers. These RNAP II phospho-modifications may also anchor elongation factors, co-transcriptional processing complexes, and chromatin-modifying enzymes

These combinations of transcription initiation factors act to kick-start proviral transcription by recruiting histone acyltransferases and chromatin remodeling enzymes whose activity on the adjacent Nuc-1 loosens up chromatin sufficiently enough to permit the recruitment of RNAP II and the formation of the preinitiation complex (Fig. 3). Specifically, NFAT or NF-κB can enhance RNAP II recruitment by directly anchoring transcriptional co-activator proteins such as CBP and p300 [181–183]. These structurally similar acetyltransferases mediate histone H3 lysine 27 acetylation (H3K27Ac). This epigenetic mark has been shown to correlate with the transition of RNAP II from initiation to elongation [184, 185]. Phosphorylation and homodimerization of STAT5 following the activation of JAK-STAT signaling also enables STAT5 to translocate into the nucleus and bind p300/CBP [185, 186]. Counteracting the SUMOylation of IL-2-induced phospho-STAT5 with benzotriazoles has been shown to enhance the occupancy of STAT5 at the HIV-1 LTR and is associated with the reactivation of latent HIV-1 in primary CD4^+^ T cells [187].

As a critical component of the preinitiation complex, TFIIH utilizes its DNA helicase activity to facilitate the transitional loading of RNAP II onto a melted short stretch of single-stranded DNA [188]. The CDK7 protein kinase subunit of TFIIH then phosphorylates the heptad repeats Y-S-P-T-S-P-S of the C-terminal domain (CTD) of RNAP II at Ser5, thereby causing the polymerase to dissociate from Mediator and facilitate its promoter escape [189]. The 26-subunit Mediator complex includes interchangeable CDK8 and CDK19 kinases, which can have both stimulatory and repressive effects depending on the gene context [190]. In a recent study, inhibition of CDK8/CDK19 kinase activity was found to suppress proviral reactivation in a Jurkat latency model and ex vivo-treated primary CD4^+^ T cells derived from virally suppressed PWH [191], but siRNA knockdown of both CDK8 and CDK19 in Jurkat cells failed to produce an effect on HIV-1 transcription, perhaps because this disrupted the integrity of the Mediator complex [192]. The Ser5-phosphorylated RNAP II CTD is also essential for recruiting the 5ʹ mRNA capping enzymes as the polymerase initiates transcription elongation [193].

The unique organization of the HIV-1 LTR and the interplay between the multiple transcription initiation factors and TFIID results in DNA bending that enhances efficient transcription initiation yet results in promoter-proximal pausing near the end of the TAR RNA hairpin found at the 5ʹ end of all HIV transcripts [194, 195]. Promoter-proximal pausing is reinforced by the negative elongation factors NELF and DSIF [195–197] and the stabilization of nucleosomal structures adjacent to the pause sites [198]. A cryoEM structure of RNAP II bound to DSIF and NELF on a DNA-RNA scaffold of the HIV-1 proximal pause site showed that pausing by NELF is allosteric [199]. Because of overlapping interaction sites, NELF and DSIF can only stabilize pausing after initiation factors are released: DSIF binding is incompatible with the binding of TFIIB and TFIIE and NELF-A is incompatible with TFIIF binding [199].

### Stimulation of promoter clearance by Tat and P-TEFb

The viral transactivator Tat and its cofactors resolve promoter-proximal pausing and enhance elongation [200, 201]. The form of P-TEFb that Tat recruits to proviral HIV-1 is a heterodimer complex of a kinase subunit CDK9 and its regulatory partner, cyclin T1 (CycT1) [202, 203]. Tat facilitates the recruitment of P-TEFb by binding directly to the nascent TAR hairpin at a U-rich bulge while CycT1 binds cooperatively to the apical loop [204–207]. Tat and P-TEFb also form a larger assembly with the super elongation complex (SEC) before cooperatively associating with the nascently synthesized TAR hairpin [208, 209] (Fig. 3).

TAR RNA binding places the catalytic CDK9 subunit of P-TEFb adjacent to the negative elongation factors on the paused polymerase complex, allowing the kinase to phosphorylate the E subunit of the repressive NELF complex at multiple serine residues, which causes NELF to dissociate from both TAR and RNAP II [196, 210]. In addition, CDK9 phosphorylates the C-terminal repeats of the Spt5 subunit of DSIF, which have high sequence homology to the RNAP II CTD sequences [211, 212]. Recent Cryo-EM structures of the paused and active elongation complexes have shown that in addition to inducing the release of NELF from RNAP II, P-TEFb kinase activity also causes a conformational change in DSIF that explains its transformation into a positive elongation factor [199, 213]. Consequently, the overall effect of the phospho-modifications by P-TEFb at the provirus is to remove the blocks to elongation imposed by NELF and DSIF and to stimulate efficient elongation and co-transcriptional processing of proviral transcripts.

Seminal studies carried out in transformed cell models showed that in the absence of Tat, RNAP II transcribing complexes on proviral HIV-1 are weakly processive. Under these conditions, short abortive transcripts of ~ 60 nucleotides accumulate in the cells due to the premature termination of transcription and release of RNAP II or an accumulation of the polymerase in a promoter-proximal region adjacent to the 59-nucleotide TAR RNA coding sequence [214–216]. Subsequently, short, prematurely terminated HIV-1 transcripts have also been detected in PBMCs obtained from both ART-treated and untreated patients [95, 217, 218], although it’s unclear what fraction of these transcripts are generated from defective proviruses. Detection of abortive HIV-1 transcripts in the absence of Tat suggests that, just like a subset of protein-coding cellular genes that include immediate early genes (IEGs), promoter-proximally paused RNAP II on proviral HIV-1 may be subjected to premature transcription termination by Integrator, a multisubunit protein complex that possesses endonuclease and phosphatase activities that are functionally analogous to those of the cleavage and polyadenylation machinery [219]. The core of Integrator binds RNAP II, DSIF, and NELF in the paused elongation complex that positions its endonuclease subunit near the RNAP II RNA exit channel to cleave nascent RNA [220, 221]. In addition, Integrator is thought to employ its protein phosphatase 2A (PP2A) subunit to remove the phospho-modifications at the C-terminal regions of both RNAP II and DSIF conferred by P-TEFb, thereby preventing the transition of paused RNAP II to productive elongation [222–224]. Whether Integrator also contributes to the early stages of elongation in the presence of Tat has yet to be investigated. Despite its ability to counteract pause release by P-TEFb, Integrator-PP2A might also be involved in mediating the transition to productive elongation by dephosphorylating pSer5 modifications at the RNAP II CTD heptad repeats, allowing enhanced CTD modification at Ser2 by Tat-activated P-TEFb. Therefore, an attractive hypothesis for the role of Integrator in regulating proviral HIV-1 transcription, consistent with its recognized role in facilitating the transcription elongation of signal-dependent genes such as IEGs [221, 225, 226], is that its dual catalytic activities enforce a dynamic turnover of RNAP II at HIV-1 promoter-proximal pause sites that is repressive but, depending on the availability of Tat and P-TEFb, can facilitate the formation of elongation-competent RNAP II complexes capable of rapidly clearing the promoter region.

Additional elongation factors also modulate RNAP II promoter-proximal pausing, which may explain why RNAP II always occupies the promoter-proximal pause site during active transcription [195]. Dissociation of NELF from RNAP II exposes a region required for binding polymerase-associated factor 1 complex (PAF1c), an elongation factor that enhances RNAP II elongation rate and processivity [213, 227–229]. Paradoxically, PAF1c also has a demonstrated role in maintaining RNAP II in a paused state at promoter-proximal regions and was identified in a siRNA high-throughput screen as a potent restriction factor for acute HIV-1 infection [230–232]. A PAF1c inhibitor can disrupt PAF1c chromatin occupancy, thereby inducing global release of promoter-proximally paused RNAP II into gene bodies and facilitating the reactivation of latent HIV-1 in both cell line models and patient-derived cells [233].

### Enhancement of Tat:P-TEFb activity by the superelongation complex

P-TEFb is an integral component of the much larger superelongation complex (SEC), which contains various combinations of 4 subunits (ELL or ELL2; EAF1; ENL or AF9; and AFF1 or AFF4) [208, 209, 234]. The SEC helps to stabilize the Tat:P-TEFb complex, promote promoter clearance, and enhance polymerase processivity. The interactions between Tat:P-TEFb and the SEC are mediated by AFF4 or its structural homolog AFF1, which are both elongated molecular scaffolds [235]. Crystal structures of the CDK9–CycT1–AFF4–Tat–TAR protein-RNA complex demonstrate the tight interface between P-TEFb, Tat, and TAR RNA [236]. The structure also revealed how stabilization of the complex by AFF4 permitted the TAR RNA central loop to engage the CycT1 TAR recognition motif (TRM) and compact core of Tat, while the extended Tat arginine-rich RNA binding motif bound to the TAR RNA major groove [236]. Additional crystal structures of the C-terminal homology domain (CHD) that is conserved among AF4/FMR2 family proteins, including AFF1, AFF2, AFF3, and AFF4, but is separated from the intrinsically disordered N-terminal region that interacts with other super-elongation complex subunits, showed that it is a substrate for CDK9, and helps trigger the release of RNAP II from the promoter-proximal pause sites [237].

The ELL or ELL2 subunits of the SEC enhance processive elongation by preventing backtracking and transcriptional arrest after pause sites [238, 239]. In resting memory T cells, ELL2 is almost absent [240], and even after TCR activation, ELL2 is restricted to rate-limiting levels due to rapid protein turnover induced by the polyubiquitination-proteasomal pathway [241]. The Tat-AFF4 complex helps to prevent the degradation of ELL2, leading to higher levels of SEC associated with P-TEFb [208, 241]. Finally, the YEATS domain of the ENL or AF9 subunit of the SEC creates additional contacts with the paused RNAP II via an interaction with the PAF1 subunit of PAFc [235], further enhancing RNAP II processivity [213, 227–229].

### Chromatin modification during elongation by Tat and P-TEFb

In addition to targeting NELF and DSIF, CDK9 also extensively phosphorylates the CTD of Rbp1, the large subunit of RNAP II, mainly at the Ser2 residues of its 52 heptad repeats [242–244]. The transient phosphorylation of Ser2 and Ser5 of the heptad repeats creates a molecular recognition code of charged residues, informally termed the CTD code [245, 246]. The phosphorylated CTD permits the recruitment of multiple factors, including splicing factors, chromatin modifying factors and additional elongation factors, required for efficient HIV transcription and post-transcriptional events. Dynamic changes in CTD phosphorylation are reflected in a gradient of Ser2 and Ser5 phosphorylation along the provirus with pSer5 enriched near the promoter and pSer2 enriched near the 3’LTR [195]. Removal of TFIIH pSer5 marks from the RNAP II CTD by the Ssu72 CTD pSer5-specific phosphatase has been shown to stimulate Tat transactivation during the early phases of transcriptional elongation [247]. Since P-TEFb cannot phosphorylate RNAP II CTD repeats carrying pSer5, dephosphorylation of Ser5 enhances CTD phosphorylation at the Ser2 and Ser7 residues [248].

The feedback communication between the CTD and histone modifications helps coordinate chromatin states with RNAP II-mediated transcription [249]. For example, by phosphorylating the linker region between the polymerase core and the CTD, P-TEFb also promotes the binding of the histone chaperone SPT6 to RNAP II at this region [213, 250]. SPT6 interacts with PAF1c and helps stabilize its presence on chromatin [251]. This recent finding aligns with the implicated role of P-TEFb activity in remodeling the chromatin barriers encountered on gene bodies by the elongation machinery during transcription.

Ser2 phosphorylation of the RNAP II CTD is also a prerequisite for the monoubiquitination of histone H2B (H2Bub1) by the E3 ubiquitin ligase complex RNF20/40, a histone modification that is associated with active transcription since it is found to be present on the transcribed regions of numerous active class II genes [252]. By contrast, the initial recruitment of P-TEFb to cellular genes requires the deubiquitination of H2B and phosphorylation of histone H3 at Ser10 (H3pS10) [253]. RNF20/40 elicits its monoubiquitination activity on H2B by binding PAF1c [254]. Subsequently, H2Bub1 stimulates efficient elongation by inducing trimethylation of histone H3 at Lys4 (H3K4me3) and methylation of Lys79 (H3K79me2/me3) by hSet1 and Dot1/Dot1L, respectively [254–257]. H2Bub1 also enhances the histone chaperone remodeling activity of FACT [258–260]. Disruption of chromatin structure by FACT via the displacement of the histone H2A/H2B dimer from core nucleosomes present in gene bodies is essential for enabling RNAP II to transcribe through nucleosomal barriers efficiently [163–166]. This interplay between H3pS10-induced P-TEFb activity on RNAP II, H2Bub1, H3K4me3, H3K79me2/me3, and chromatin remodeling by the histone chaperones SPT6 and FACT is likely to affect the positioning of Nuc1 and Nuc2 on the HIV provirus and further help stimulate processive HIV-1 transcription.

### Tat-mediated delivery of P-TEFb to proviral HIV-1

**Fig. 4:**
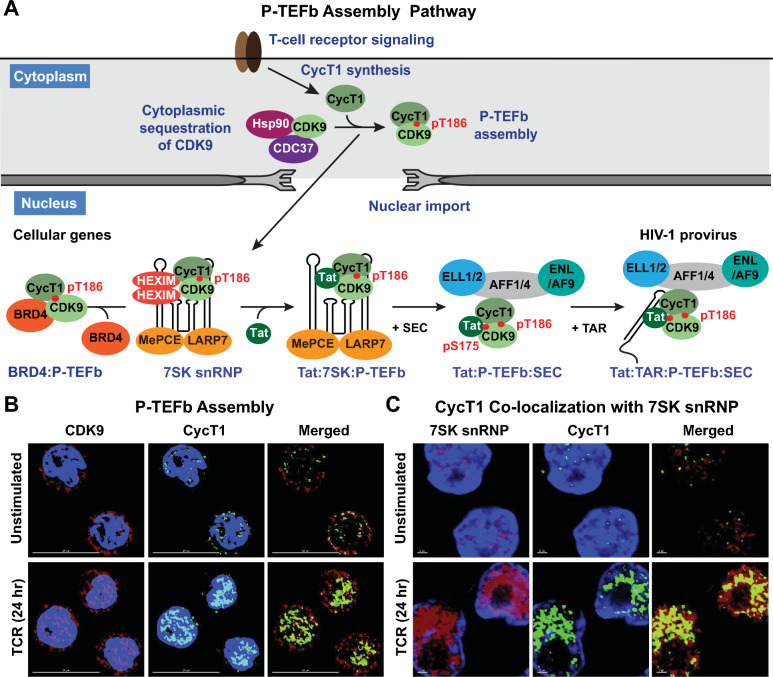
Biogenesis of P-TEFb in primary T cells and proposed mechanism for the recruitment of Tat:P-TEFb to the HIV-1 provirus. **A** P-TEFb is expressed in resting memory CD4^+^ T cells at vanishingly low levels due to posttranscriptional mechanisms that limit CycT1 expression. This causes the CDK9 subunit to be sequestered in the cytoplasm by the kinase-specific chaperone complex Hsp90/Cdc37. TCR co-stimulation induces CycT1 protein synthesis, leading to the heterodimeric assembly of P-TEFb that is stabilized by CDK9 phosphorylation at Thr186. Upon assembly, P-TEFb then enters the nucleus where it is incorporated into the 7SK snRNP complex. BRD4 and Tat can physically engage with 7SK snRNP but compete with one another to dissociate P-TEFb from the complex. The signal-dependent modification pSer175 CDK9 preferentially enhances the binding of Tat to P-TEFb. Ser175 on the activation loop of CDK9 is an essential contact point for BRD4; mutation of Ser175 to a phosphomimetic residue or an alanine produces severe defects in the association of P-TEFb with BRD4. **B** Microscopic imaging of the assembly of P-TEFb in memory CD4^+^ T cells following TCR activation. Cells were stimulated or not with soluble anti-CD3 and anti-CD28 antibodies. After immunostaining, images were captured by deconvolution microscopy at 60x. Scale bar: 10 μm. CDK9 is sequestered in the cytoplasm in the unstimulated cells, and CycT1 is present in very low amounts. After stimulation, CDK9 and synthesized CycT1 are present in the nucleus as a complex (P-TEFb), presumably sequestered by 7SK snRNP. **C** Combined immunofluorescence and RNA FISH detection of CycT1 and 7SK snRNA in memory CD4^+^ T cells. Cells were stimulated or not with anti-CD3 and anti-CD28 Dynabeads at a 1:1 bead-to-cell ratio. Images were captured by deconvolution microscopy at 100x. Scale bar: 1 μm. Cellular stimulation results in a doughnut-shaped redistribution of 7SK snRNA within the nucleus that tightly colocalizes with induced CycT1

In actively dividing cells, including transformed T cells, at least half of the P-TEFb complexes are reversibly associated with 7SK snRNP in the nucleus [261, 262] (Fig. 4). 7SK snRNP comprises a molecule of the 331-nucleotide long non-coding 7SK snRNA that is protected at its 5’ and 3’ ends from exoribonucleolytic degradation by the capping enzyme MEPCE and the La-related protein LARP7, respectively [263–266]. After RNA capping, MEPCE remains bound to the 5ʹ monomethyl phosphate cap and forms direct interactions with LARP7, resulting in a closed loop conformation of the RNA that stabilizes the ‘core’ 7SK snRNP structure [266–268]. In this conformation, the three-dimensional folding of 7SK snRNA establishes a molecular scaffold that cooperatively binds two molecules of P-TEFb to a homodimer of the proteins HEXIM1 or HEXIM2, which are thought to inhibit CDK9 kinase activity by directly occluding the enzyme’s active site [269, 270]. Bulk RNA-seq transcriptome analysis of various primary CD4^+^ T cell subsets showed that HEXIM1 mRNA is expressed at approximately four and sevenfold higher levels than HEXIM2 in unstimulated and activated cells, respectively, suggesting that HEXIM1 is the major isoform in CD4^+^ T cells and thus the more likely regulatory partner for P-TEFb within 7SK snRNP [240].

P-TEFb is recruited to most cellular genes via BRD4, a bromodomain-containing protein that binds to acetylated histones and directs P-TEFb to activated cellular genes [271, 272]. To be able to recruit P-TEFb to proviral HIV-1 effectively, Tat must compete with BRD4, which uses its two tandem N-terminal bromodomains to bind acetylated histones H3 and H4 (AcH3 and AcH4) and contains a well-defined C-terminal P-TEFb-interacting domain (PID) sufficiently capable of dissociating P-TEFb from 7SK snRNP through as of yet unclear mechanisms [271, 273, 274]. The BRD4 PID can also inhibit Tat-dependent HIV-1 transcription, most likely by acting as a decoy for P-TEFb binding [275].

Like BRD4, Tat has evolved the molecular ability to physically engage with 7SK snRNP and remove P-TEFb from the complex [276]. However, unlike BRD4, Tat is known to form well-defined interactions with the 7SK snRNA scaffold [277, 278]. Several models have been proposed to explain the process of Tat-dependent extraction of P-TEFb, including (a) assembly of Tat and 7SK snRNP at the HIV-1 promoter and competitive displacement of Tat:P-TEFb from 7SK snRNP by nascent TAR RNA [279, 280]; (b) competitive dislodgement of HEXIM1 from 7SK snRNA via Tat interactions with the stem region of the 5ʹ hairpin leading to the formation of a Tat:7SK snRNA:P-TEFb intermediate that mediates exchange of Tat:P-TEFb to the SEC (Fig. 4) [277, 278, 281–283]; (c) engagement of promoter-bound 7SK snRNP by PPM1G to catalyze Thr186 dephosphorylation of CDK9 thereby facilitating the displacement of P-TEFb and its exchange onto Tat [284]; and (d) recruitment by Tat of a ubiquitin ligase UBE2O to 7SK snRNP that ubiquitinates HEXIM1 leading to 7SK snRNP disruption, cytoplasmic sequestration of HEXIM1 and enhancement of the fraction of P-TEFb that is associated with Tat [285].

Observations from recent biochemical and structural studies demonstrating that Tat and HEXIM1 must interact with 7SK snRNA in a mutually exclusive manner suggest that there may be the formation of a Tat:7SK snRNA:P-TEFb intermediate. The recognition of the 5’ hairpin of 7SK by the arginine-rich RNA binding domain (RBD) of Tat is enabled by Arg52, which remodels a pseudo arginine sandwich motif critical for HEXIM1 binding into a classical arginine sandwich motif (ASM), leading to the displacement of HEXIM1 [277, 278]. Analogously, to efficiently interact with TAR RNA, Tat also utilizes Arg52 to remodel the U-rich bulge near the top of the stem of the TAR hairpin into an ASM whose structure is identical to that of the remodeled ASM in 7SK [206, 278]. Moreover, NMR structural analysis of the interaction of the HEXIM1 RNA binding motif with the apical stem portion of the 7SK 5’ hairpin reveals that in addition to exhibiting a three to fourfold lower binding affinity compared to Tat, HEXIM1 induces a local destabilization of this region that Tat further exploits to bind 7SK more efficiently [277]. While significant strides have been made in delineating the mechanism of Tat:7SK assembly, how Tat:P-TEFb is eventually dislodged from 7SK and directed to TAR RNA anchored at the proviral locus is less well understood. Whether this exchange process in vivo requires the mediation of TAR, the presence of the SEC scaffolding components AFF1/AFF4, is facilitated by specific PTM switches, or can be sufficiently accomplished by Tat requires unequivocal clarity considering that it is a crucial intermediary step in proviral HIV-1 gene regulation.

### Tat interactions with chromatin-modifying enzymes

More than two decades ago, a number of groups documented that Tat mediates the recruitment of the histone acetyltransferases (HATs) p300/CBP, hGCN5, and PCAF as well as the SWI/SNF remodeling complex to the proviral LTR [127, 286–292]. The HATs contribute indirectly to Tat’s transactivator function by changing the chromatin environment and directly by modifying Tat. In addition to targeting Nuc-1, HATs directly modify Tat on Lys50 and Lys51, which are situated within the Arginine-rich TAR RNA binding motif (ARM) [290, 293, 294]. Acetylation of the ARM region dissociates Tat from TAR while simultaneously reinforcing its interaction with chromatin-modifying transcriptional co-activators (both HATs and SWI/SNF subunits) by providing acetyl binding sites for their bromodomains [291, 295]. Consequently, these ARM acetyl modifications provide a bridge to interact with RNAP II and facilitate a TAR-independent co-transcriptional remodeling of the chromatinized proviral template during the elongation phase of transcription [296, 297]. By contrast, PCAF binding to acetylated Lys50 of Tat allows it to acetylate Lys28 situated within the cysteine-rich region of Tat’s activation domain, leading to the abrogation of Tat:PCAF interactions and enhancement of the association of Tat with P-TEFb that stabilizes the Tat:P-TEFb:TAR ternary complex [293, 295, 298, 299].

Tat post-translational modifications also mediate other phases of transcription. At the end of the transcription cycle, SIRT1 is thought to bind and deacetylate Tat at Lys50, thereby recycling Tat for subsequent rounds of viral transcription [300]. Following the deacetylation of Tat at the ARM region, its monomethylation at Lys51 by Set7/9 bound to TAR has been reported to enhance Tat-dependent HIV-1 transcription by restoring Tat interactions with TAR necessary for the recruitment of P-TEFb [301]. A recent study has also identified the lysine methyltransferase SMYD5 as an additional co-activator of HIV-1 transcription in cell line models [302]. SMYD5 can be recruited to cellular genes by RNAP II and catalyzes the trimethylation of histone H3 at Lys 36 (H3K36me3), a modification associated with actively transcribing genes [303]. SMYD5 is also capable of modifying Tat and binding TAR in in vitro assays [302]. Although the interactions with Tat were found to promote the intracellular expression of SMYD5 [302], the site of Tat methylation by SMYD5, as well as its functional implication in the epigenetic regulation of HIV-1 transcription, remain to be determined. Finally, demethylation of Lys51 by LSD1/CoREST allows for the re-acetylation of the ARM, which triggers re-entry into the TAR-independent phase of transcription elongation [304].

How all the complex interactions between Tat, histone modifying enzymes, and the super elongation complex (SEC) are coordinated at TAR to affect the transactivation of HIV-1 remains unclear. It will be worthwhile to generate epitope-specific antibodies directed towards the identified post-translational modifications of the ARM and activation domain regions of Tat to examine the kinetics of their acquisition and loss of the major PTMs and their functional relevance in primary cell latency models. Recently, the efficient reversal of HIV-1 latency in CD4^+^ T cells from PWH due to the ectopic expression of Tat introduced by transduction with lipid nanoparticles containing Tat mRNA has been reported [305]. This activity might be due to the multifunctional transactivator roles of Tat in recruiting HATs to the proviral promoter and coordinating the delivery of P-TEFb and SEC to the promoter-proximally paused RNAP II at the TAR hairpin. However, since Tat can also stimulate TNF-α IL-1β and other proinflammatory cytokine production in T-cells and myeloid cells [306–308], some of the latency-reversing effects of Tat might also be due to indirect effects of the cytokines leading to enhanced T-cell activation and P-TEFb production.

### Stochastic transcription from the viral LTR

One important consequence of the promoter-proximal pausing at the HIV-1 LTR is that HIV transcription at the level of an individual provirus occurs in rapid bursts [309, 310]. The stochastic switching of the viral promoter between ON and OFF states is caused by random binding dynamics of the core transcription factors TBP, Sp1, and NF-κB, which can lead to intermittent clearance of the paused RNAP II complexes [311]. Elegant single-molecule imaging of HIV-1 transcription has shown that RNAP II enters a long-lived pause when HIV-1 Tat is limiting (> 20 min), effectively limiting viral transcription [312]. Consistent with the mechanisms described above, promoter clearance is restricted in the absence of Tat, although periodically, the viral genome can be transcribed in brief pulses containing 10 or more RNAP II complexes. By contrast, when Tat is present in saturating concentrations, RNAP II rapidly clears the promoter region (one every 7–15 s), and forms convoys of elongating transcription complexes [312].

One caveat to these experiments is that they were performed in transformed cell models where epigenetic silencing is probably less effective than in primary cells. Another key difference is that in the latently infected primary cells, the promoter recruitment of transcription initiation factors is rate-limiting. However, chromatin immunoprecipitation experiments have also indicated that promoter-proximal pausing of RNAP II appears to be an essential regulatory feature of the transcriptional control of HIV-1 in primary CD4^+^ T cell models of HIV latency [21, 313]. As in the transformed cell model systems, Tat also facilitates HIV-1 promoter clearance in infected primary CD4^+^ T cells.

Indirect evidence for RNAP II promoter-proximal pausing in primary cells stems from the observations of short, terminated HIV-1 transcripts approximately the size of TAR RNA detected in PBMCs obtained from both ART-treated and untreated PWH [217]. Short transcript levels are strongly associated with an existing chronic state of immune activation as defined by the co-expression of HLA-DR and CD38 on CD8^+^ T cells [217]. Additional evidence of viral transcription elongation control in latently infected primary CD4^+^ T cells comes from two recent studies that utilized a reverse transcription droplet digital PCR assay (RT-ddPCR) [218, 314]. Using these methods, short TAR-containing transcripts were detected in blood CD4^+^ T cells and there was a significant post-initiation restriction to proviral transcription [218]. These blocks could be reversed by either TCR activation or stimulation with the protein kinase C (PKC) agonist ingenol-3-angelate, which stimulated the completion of viral RNA synthesis and accumulation of polyadenylated and multiply spliced viral transcripts [218]. By contrast, in T cells obtained from rectal biopsies, there was clear evidence of a block to proviral initiation and, to a significantly lesser extent, blocks to proviral elongation and RNA processing [314]. An important limitation of these studies was that it was impossible to distinguish between cells carrying replication-competent and defective viruses, raising the concern that most of the abortive transcripts seen in the PBMC samples arose from defective proviruses. Nonetheless, identifying tissue-specific differences in the mechanisms governing the transcriptional regulation of latent HIV-1 will be an essential consideration when designing latency reversal strategies to disrupt the HIV-1 reservoir in its various tissue compartments [314].

### Tat-independent proviral transcription

Transcription elongation of latent proviruses is inefficient in the absence of Tat but necessary to kick-start the formation of multiply spliced transcripts required for Tat and Rev synthesis. The processes and factors supporting the earliest cycles of proviral transcription elongation remain unclear, and there are numerous potential mediators for P-TEFb and SEC recruitment to the HIV provirus. First, Tat-independent recruitment of the SEC to promoter-proximally paused RNAP II on latent proviral HIV-1 might be achieved via an interaction between the conserved YEATS domain of the adaptor proteins ENL or AF9 and the PAF1 subunit of PAF1c [235]. In this scenario, since ELL2 mRNA and protein expression are highly restricted in resting T cells, possibly due to transcriptional repression and rapid protein turnover [240, 241], ELL may likely substitute for its elongation activity. Generation of Tat in activated T cells would then coincide with the inducible synthesis of ELL2, at which point Tat and the SEC scaffold protein AFF1/AFF4 can promote the assembly of ELL2-containing SEC complexes by preventing the rapid proteasomal degradation of ELL2 [241]. Ultimately, by stabilizing the expression of ELL2, Tat could significantly enhance the fraction of P-TEFb associated with the SEC [208].

Another plausible theory is that the initial recruitment of P-TEFb to latent proviral HIV-1 might be effected by the bromodomain-containing protein BRD4. Interestingly, while nucleosomes occupying the LTR region of latent HIV-1 have been found to contain low AcH3 levels, they characteristically possess elevated AcH4 levels due to KAT5 acetyltransferase activity that permits the occupancy of this chromatin region by both the long and short isoforms of BRD4 [315]. The short isoform of BRD4 lacking the C-terminal PID can promote HIV-1 latency by mediating the recruitment of repressive BAF SWI/SNF complexes [125]. By contrast, although the long isoform of BRD4 is also well-understood to be a potent suppressor of Tat-dependent transactivation of HIV-1 [272, 275, 316], its presence on latent proviruses could aid in the initial exchange of P-TEFb from 7SK snRNP complexes that have been recruited *en bloc* to the HIV-1 LTR. The enzymatic activities of BRD4, including its histone acetyltransferase activity, may also enhance its ability to stimulate RNAP II activity in the absence of Tat [317, 318]. A recent study utilizing bromodomain deletion mutants of BRD4 has demonstrated that the C-terminal PID of BRD4 is not only sufficient for P-TEFb binding but can also stimulate the genome-wide release of RNAP II from promoter-proximal pausing [319]. This interesting observation suggests that while the two tandem bromodomains are essential for maintaining the residence of BRD4 on genic regions, particularly at or near transcription start sites, in part to preserve the transmission of epigenetic memory during cell division [320, 321], BRD4 can also deliver P-TEFb to the paused RNAP II complex without being tethered to chromatin via its bromodomains. Whether BRD4 needs to be dissociated from chromatin to recruit P-TEFb from 7SK snRNP and how the BRD4:P-TEFb complex is then linked to the hypophosphorylated RNAP II for P-TEFb to elicit its CTD kinase activity are important questions that remain to be addressed.

Activators of transcription initiation have also been postulated to mediate P-TEFb recruitment. For example, NF-κB was initially proposed to directly recruit P-TEFb to the proviral LTR [322]. However, more recent studies have identified several more likely candidate auxiliary factors that either directly recruit P-TEFb or do so indirectly by binding 7SK snRNP. These include the cellular stress response protein HSF1, TRIM24 (aka TIF1α), TRIM28 (aka. KAP1 or TIF1β), and the SR protein splicing factors SRSF1 and SRSF2 (aka. SC35) [323–327].

HSF1 activity as a positive HIV-1 transcription elongation factor requires a concomitant induction of P-TEFb biogenesis in primary T cells and is bolstered by immunoprecipitation experiments conducted in the J-Lat cell line latency model showing that HSF1 can form a complex with P-TEFb that can directly bind the HIV-1 LTR [328]. Inhibition of HSF1 was found to effectively suppress the formation of elongated and processed viral transcripts in PMA/ionomycin-treated patient-derived CD4^+^ T cells as measured by RT-ddPCR assays for HIV-1 transcription elongation [323]. By contrast, HSF1 inhibition did not affect the appearance of short, TAR-inclusive transcripts that serve as a readout of successful viral transcription initiation. Interestingly, HSF1 inhibition was also ineffective at suppressing the formation of elongated viral transcripts in cells stimulated through the TCR, suggesting that HSF1 may play an essential role in mediating the stimulation of HIV-1 transcription elongation in response to inducers of cellular stress but not physiological T-cell signaling [323].

Through its interaction with the transcription factor complex RBF-2 that binds Ras response enhancer elements, TRIM24 has recently been shown to mediate the signal-dependent recruitment of P-TEFb to the proviral LTR in Jurkat T cells [324]. However, whether the recruitment of P-TEFb by TRIM24 can occur in the absence of Tat is unclear. Finally, TRIM28 is a transcription factor linked to both the activation and repression of a subset of cellular genes and proviral HIV-1 [325, 329–331]. In latently infected Jurkat E4 cells, TRIM28 has been shown to bind and recruit the P-TEFb regulatory complex 7SK snRNP to the proviral promoter under basal and stimulatory conditions [325]. This finding suggests that P-TEFb exists on latent HIV-1 as a complex with 7SK snRNP at or near the promoter-proximal region where RNAP II is transcriptionally paused. This scenario is plausible since 7SK snRNP functions to repress CDK9 kinase activity.

It is important to note, however, that recruitment of the 7SK snRNP complex to the provirus is insufficient to stimulate transcription since this requires P-TEFb to be exchanged from the promoter-bound 7SK snRNP to the elongating RNAP II, a process that likely is to be stimulus-dependent. The most attractive candidate factors that may mediate P-TEFb exchange in the absence of Tat are the SR splicing factors SRSF1 and SRSF2. SRSF1 binds to TAR RNA at a region that overlaps with the Tat recognition site and can inhibit Tat transactivation of proviral HIV-1 by directly competing for its binding to TAR while also increasing the basal level of HIV-1 transcription [327]. SRSF2 is widely regarded as a marker of nuclear speckles, where 7SK snRNP is known to accumulate [332]. SRSF2 reportedly associates with 7SK snRNP by binding the third stem-loop of 7SK snRNA and recruits the RNP to cellular genes where SRSF2 binds nascently synthesized RNA to mediate the transfer of P-TEFb to promoter-proximally paused RNAP II in a manner analogous to the recruitment of P-TEFb to TAR by Tat [326]. Consistent with this finding, depletion of SRSF2 enhanced RNAP II pausing within cellular gene bodies, impaired P-TEFb recruitment, and decreased polymerase processivity [333]. Although SRSF2 can recognize highly degenerate mRNA sequence elements that are proximal to transcription start sites and does not appear to require specific secondary motifs, it is unknown whether it can interact with HIV-1 TAR sequences. Thus, although, logically, some basal transcription takes place to initiate Tat synthesis, the exact mechanism to achieve this in latently infected cells remains uncertain.

### Induction of transcription initiation by TCR-signaling pathways

There are no virus-specific mechanisms that mediate HIV-1 latency. Instead, HIV-1 latency in memory CD4^+^ T cells results from the nuclear exclusion of transcription initiation factors and vanishingly low expression of the transcription elongation factor P-TEFb, which constitute the two primary blocks to HIV-1 transcription in memory CD4^+^ T cells [334]. In addition, as described above, HIV-1 latency is reinforced, even in replicating cell systems, by restrictive epigenetic factors accumulating at the proviral promoter,

In ex vivo primary cell models or in latently infected T cells recovered from donors, TCR co-stimulation with anti-CD3 and anti-CD28 antibodies is the most effective at reactivating latent HIV-1 since it provides all the intracellular signals necessary to induce the nuclear mobilization of key transcription initiation factors, stimulate the posttranscriptional biogenesis of P-TEFb, and counteract epigenetic silencing [240]. By contrast, proinflammatory cytokines released by either activated CD4^+^ T helper 1 or 2 cells or macrophages, including IFN-γ, TNF-α, IL-2, and IL-15, are much weaker activators of P-TEFb and are therefore unable to stimulate processive HIV-1 transcription in primary cell latency models [240, 335].

**Fig. 5:**
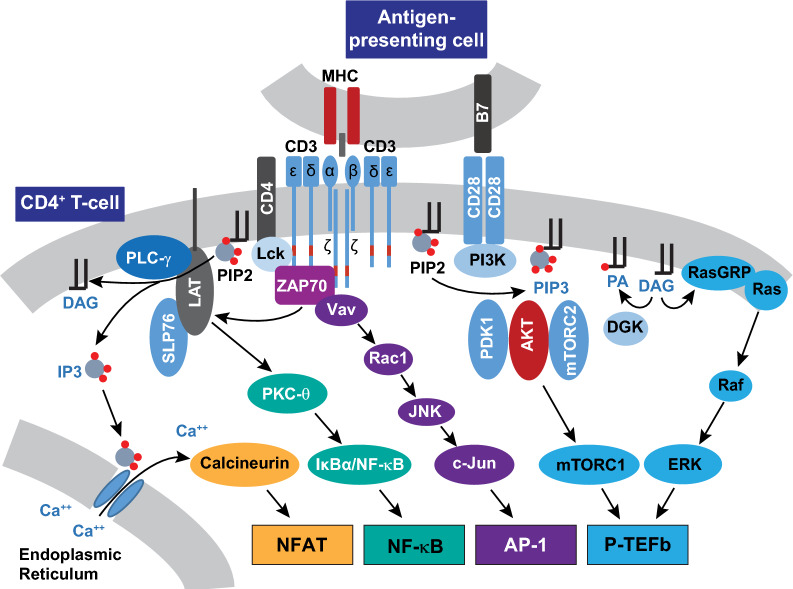
Membrane events and generation of intracellular second messengers essential for TCR-induced mobilization of activators of transcription initiation and P-TEFb. During antigen presentation, maximal activation of TCR signaling requires a costimulatory engagement that usually involves a pairwise linkage between B7 and its T-cell counterpart CD28. TCR stimulation triggers a receptor and non-receptor phospho-tyrosine cascade that results in the generation of diacylglycerol (DAG), intracellular mobilization of calcium (Ca^++^) from the endoplasmic reticulum (ER), and formation of phosphatidylinositol-3,4,5 triphosphate (PIP_3_) which serves to anchor PDK1, AKT, and mTORC2 to the membrane. PLCγ-generated DAG initiates the membrane recruitment and activation of PKC-θ and RasGRP. DAG can be phosphorylated by diacylglycerol kinase (DGK), which leads to the curtailing of DAG signaling and the generation of an additional lipid second messenger phosphatidic acid (PA)

Initiation of TCR signaling in CD4^+^ T cells in vivo usually occurs in the context of an antigen-presenting cell (APC) presenting pathogen-derived peptides bound to MHC Class II transmembrane proteins on the cell surface (Fig. 5). For maximal T-cell activation to be enabled, binding of the peptide-MHC complex to the TCR complex is accompanied by a costimulatory engagement by the APC. This typically involves a pairwise linkage between the integral membrane protein B7 from the APC and its T-cell counterpart, CD28 [336].

**Fig. 6:**
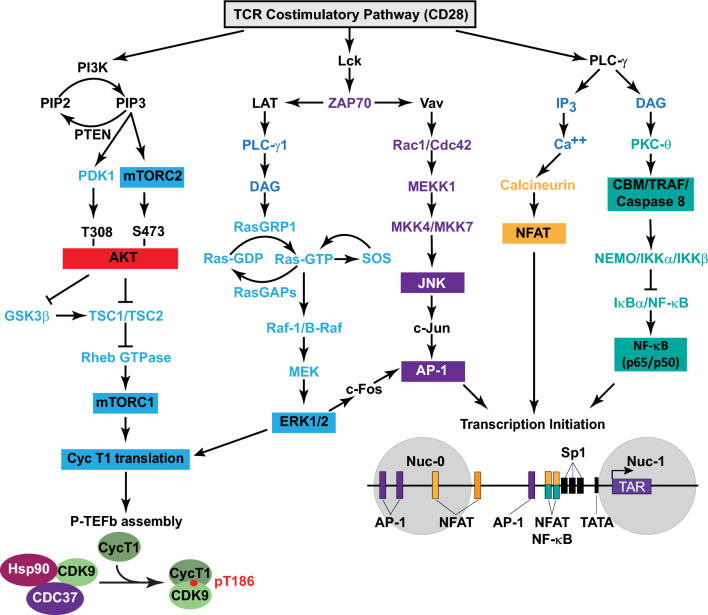
TCR signaling pathways in primary CD4^+^ T cells contribute to the stimulation of proviral HIV-1 transcription. RasGRP1-Ras-Raf-MEK-ERK1/2 and PI3K-mTORC2-AKT-mTORC1 complement one another in stimulating the posttranscriptional synthesis of CycT1, leading to P-TEFb assembly. The exact mechanisms by which ERK and mTORC1 stimulate CycT1 translation are yet to be fully delineated. Although intracellular calcium release, the activation of PKC-θ and the JNK MAPK pathway are dispensable for the formation of P-TEFb, they are likely to mediate the recruitment of RNA polymerase II (RNAP II) to proviral HIV and thus its eventual phosphorylation by P-TEFb to stimulate processive transcription elongation

TCR activation initiates three complementary signaling cascades that lead to initiation factor mobilization and P-TEFb biogenesis. First, TCR co-stimulation immediately induces a tyrosine phosphorylation cascade at the cell membrane that leads to the activation of the lipid metabolizing enzymes phosphoinositide-3-kinase (PI3K) and phospholipase C-γ (PLC-γ) [337], as diagrammed in Figs. 5 and 6. Class I PI3K enzymes, which are usually constitutively bound to CD28 at the T-cell plasma membrane, act to phosphorylate phosphatidylinositol-4,5-bisphosphate (PIP_2_) to form phosphatidylinositol-3,4,5-trisphosphate (PIP_3_) upon activation. PIP_3_ then anchors a wide range of signaling proteins through their pleckstrin homology (PH) domains [338–340]. The binding of PIP_3_ to the PH domain of SIN1, an essential and unique component of the mTOR-containing complex mTORC2, releases the inhibitory interaction between the SIN1 PH domain and the mTOR kinase domain, leading to mTORC2 activation [341].

PIP_3_ also binds the PH domains of PDK1 and AKT. PIP_3_ binding to AKT induces a conformational change that allows PDK1-mediated phosphorylation of the activation loop segment of AKT at Thr308 [342]. Full activation of AKT by PI3K-mediated signaling is achieved by mTORC2 phosphorylation of AKT at Ser473 [343]. Ultimately, downstream signaling from AKT results in the activation of a second mTOR-containing complex, mTORC1, widely considered to be central to the stimulation of ribosomal activity [344, 345], and which we have recently found is an essential regulatory component of the biogenesis of P-TEFb [240] (Fig. 6).

In the second complementary pathway, PLC-γ anchored to the membrane by binding PIP_3_ via its PH domain and activated by tyrosine phosphorylation of its Src Homology 2 (SH2) domain [346, 347], hydrolyzes PIP_2_ to generate the second messenger inositol-1,4,5-triphosphate (IP_3_), which mobilizes calcium release from the endoplasmic reticulum into the cytoplasm. Therefore, the activation of PI3K through the co-stimulatory receptor CD28 can likely contribute to PLC-γ PH domain membrane targeting and PLC-γ activation. PIP2 hydrolysis also generates diacylglycerol (DAG), which directly contributes to the activation of several C1 domain-containing intracellular signaling proteins including protein kinase C (PKC) enzymes and the Ras guanine nucleotide exchange factor RasGRP [348–350] (Figs. 5 and 6). Elevated cytoplasmic calcium levels stimulate the phosphatase calcineurin, resulting in the activation and nuclear translocation of NFAT, the transcription factor that is essential for mediating the initiation of proviral HIV-1 transcription in primary CD4^+^ T cells [21, 22, 167].

C1 domains are approximately 50 amino acids long, are cysteine-rich, and contain twin zinc fingers that coordinate proper domain folding [348]. Structural studies have shown that DAG binding to a hydrophilic cavity in the C1 domain results in a contiguous hydrophobic surface that enables the region to interact favorably with the lipid bilayer, thereby stabilizing PKC and RasGRP membrane insertion [351, 352]. Of the four known isoforms of RasGRP, the C1 domains of RasGRP1, RasGRP3, and RasGRP4 bind DAG or DAG-mimicking phorbol esters with high affinity and, consequently, they get recruited to the plasma membrane where they can access membrane-anchored Ras to facilitate guanine nucleotide exchange [350, 353].

DAG binding to the C1 domains of conventional and novel PKC isoenzymes leads to their membrane recruitment and stimulation of their kinase activities [349]. Of these PKC enzymes, PKC-θ is the predominant functional isoform in T cells and is critical for mediating both TCR-induced NF-κB and global T-cell activation [354–356]. Indeed, the reactivation of latent HIV-1 in primary cells by DAG-mimicking PKC agonists is mainly due to their ability to stimulate the activity of the canonical NF-κB transcription factor complex p65 (Rel A):p50 through PKC-θ [167, 240].

Finally, TCR signaling activates the JNK and ERK MAPK signaling pathways which lead to the assembly and nuclear induction of c-Fos:c-Jun AP-1 complexes (Fig. 6). The cooperative interaction between AP-1 and NFAT is essential for HIV-1 and IL-2 gene transcription [357]. The MAPK signaling pathways are initiated by the stimulation of RasGRP1 by DAG, which in T cells is required for the allosteric activation of SOS, a second Ras GDP-GTP exchange factor that is directly anchored to the membrane by a complex of tyrosine-phosphorylated LAT and the adaptor protein Grb2 [358, 359]. Guanine nucleotide exchange activity of SOS is triggered by the initial activation of Ras GTPase by RasGRP1 through a positive feedback mechanism that involves the allosteric binding of GTP-bound Ras to SOS [358]. In turn, activation of Ras by RasGRP and SOS results in a conformational change in Ras, which enables the robust formation of Ras:Raf complexes at the membrane that eventually leads to the activation of the MAP kinase isoforms ERK1 and ERK2 [360]. By contrast, c-Jun N-terminal kinase (JNK) MAPK signaling is a stress-responsive pathway whose activation is mediated through stimulation of Rac1 GTPase by the GDP-GTP exchange factor Vav [361]. Upon TCR co-stimulation, Vav is anchored to the membrane by utilizing its SH2 (Src Homology 2) domain to bind tyrosine-phosphorylated ZAP-70 [362, 363], eventually leading to the assembly and nuclear induction of c-Fos:c-Jun AP-1 complexes in part elicited by Vav-Rac1-MEKK1-MKK4/7-JNK-c-Jun signaling.

### Regulation of P-TEFb by TCR signaling

In primary T cells, P-TEFb expression is enabled by TCR signaling in parallel with the nuclear mobilization of transcription initiation factors. The biogenesis of P-TEFb is illustrated in Fig. 4. Immunoprecipitation studies have clearly shown that 7SK snRNP is largely unassembled in resting memory T cells due to restricted CycT1 expression and Thr186-dephosphorylation of the activation loop of CDK9 [364]. High-resolution imaging experiments employing a combination of immunofluorescence and RNA FISH have indicated that 7SK snRNP assembly occurs within a few hours after TCR activation concomitant with the nuclear localization of HEXIM1 [240]. In these imaging studies, the 7SK snRNA gradually forms a doughnut structure surrounding the nucleolus that colocalizes with both nucleoplasmic P-TEFb (Fig. 4) and the Ser2 CTD-phosphorylated RNAP II (Mbonye and Karn, unpublished results) as reactivation progresses. Thus, assembled 7SK snRNP in activated memory T cells appears to be situated within the same subnuclear phase-separated condensates that harbor transcriptionally poised genes, including proviral HIV-1, perhaps to enable the convenient exchange of P-TEFb from 7SK snRNP.

Most circulating memory CD4^+^ T cells in a healthy adult are in a quiescent state characterized by the absence of cell proliferation markers (such as Ki67 and cell cycle cyclin proteins) and the nuclear exclusion of critical transcription activators [240]. These resting T cells are also deficient in P-TEFb due to a posttranscriptional block of CycT1 mRNA translation combined with the proteasomal degradation of the CycT1 protein [365–369]. The pre-existing CycT1 transcripts in resting memory T cells may be restricted to cytoplasmic ribonucleoprotein condensates, such as stress granules, that are known to harbor translationally repressed mRNAs [370].

Biochemical and imaging studies of quiescent T cells have shown that in the absence of CycT1, CDK9 is sequestered in an inactive form in the cytoplasm by the kinase-specific chaperone complex Hsp90/Cdc37 [240, 371, 372]. Stimulation of TCR signaling induces the posttranscriptional synthesis of CycT1, which triggers the exchange of CDK9 from Hsp90/Cdc37 and the phosphorylation of CDK9 on its activation loop at Thr186 (pThr186 CDK9) [240, 364, 371]. Subsequently, pThr186 CDK9 coordinates the stable heterodimeric assembly of P-TEFb, which may be a prerequisite for the enzyme’s import into the nucleus [371, 373]. Coordination of P-TEFb assembly by pThr186 CDK9 is also required to incorporate P-TEFb into 7SK snRNP [261], which acts as an exchange pool for delivering P-TEFb to genomic sites of active transcription [269, 374–376].

An additional inducible phosphorylation site on the activation loop of CDK9, pSer175 CDK9, identified by tandem mass spectrometry, is only present on a subset of P-TEFb that is dissociated from 7SK snRNP [272]. Mutation of Ser175 to a phosphomimetic residue or an alanine produces severe defects in the association of the P-TEFb heterodimer CDK9:CycT1 with BRD4 [271, 272]. These findings indicate that the unmodified Ser175 is essential for mediating a critical interaction between the activation loop region of CDK9 and the C-terminal P-TEFb interacting domain of BRD4. We have also demonstrated that Tat can coopt pSer175 CDK9 to enhance Tat’s interaction with P-TEFb to facilitate the competitive recruitment of P-TEFb to proviral HIV-1 [272]. Based on these observations, we have developed an immunofluorescence flow cytometry assay that defines the formation of transcriptionally active P-TEFb as the dual detection of CycT1 and pSer175 CDK9 [240]. This immunofluorescence flow assay (P-TEFb immuno-flow) has proven to be valuable in enabling the identification of stimuli and small molecule agents that can induce active P-TEFb expression in primary CD4^+^ T cells as well as permitting the investigation of signaling pathways that mediate their stimulatory effects.

Of all the stimuli tested thus far by P-TEFb immuno-flow, TCR co-stimulation is the most robust at eliciting active P-TEFb expression in memory CD4^+^ T cells. By dissecting the complex array of TCR signaling pathways in inhibitor-based experiments in a primary T-cell model of HIV-1 latency [21, 377] alongside healthy donor-derived memory T cells, we have been able to define the signaling pathways that are essential for the generation of transcriptionally active P-TEFb and P-TEFb-dependent proviral reactivation [240]. Crucial among these findings is the demonstration that the RasGRP1-Ras-Raf-MEK-ERK1/2 and PI3K-mTORC2-AKT-mTORC1 pathways can complement one another in inducing the posttranscriptional elevation of CycT1 [240]. Combined inhibition of these pathways with a PI3K inhibitor (LY294002) and the MEK inhibitor, U0126, abrogates active P-TEFb expression and substantially suppresses latent HIV-1 reactivation. PI3K-mTORC2-AKT-mTORC1 affects P-TEFb biogenesis, most likely by facilitating the translation of CycT1 (Fig. 4). This is consistent with the notion that mTORC1 is well established to be essential for the initiation of protein synthesis by triggering the sequential phosphorylation of components of the translation machinery, including the ribosomal protein S6 kinase (S6K), its downstream substrate ribosomal protein S6 (rpS6) as well as translation initiation and elongation factors [345]. Since DAG-mimicking C1 domain agonists are not known to signal through PI3K or AKT, it will be interesting to determine whether their stimulation of ERK1/2 activity through RasGRP1 can lead to P-TEFb biogenesis through the activation of mTORC1 or by inducing a similar phosphorylation cascade of translation factors.

Several studies have demonstrated that the dissociation of 7SK snRNP to release the elongation activity of P-TEFb can occur in response to stress signals or activation of specific cellular pathways, including calcium, PKC-θ, ERK MAPK, and PI3K/AKT signaling [262, 378–381]. For instance, using a Jurkat T-cell model system of HIV-1 latency, we have shown that a brief (within one h) challenge of these cells with a DAG-mimicking phorbol ester or through the TCR is sufficient to cause the release of P-TEFb from 7SK snRNP in an ERK MAPK-dependent manner [262]. By conducting chromatin immunoprecipitation studies, we demonstrated further that inhibition of ERK activation substantially suppresses HIV-1 transcription elongation by preventing the recruitment of P-TEFb to the TAR region of proviral HIV-1 [262]. These findings have generated the hypothesis that the signal-dependent release of P-TEFb from 7SK snRNP is due to changes in RNA and post-translational modifications (PTMs) that trigger conformational changes that disrupt the assembly of the ribonucleoprotein complex. Subsequently, certain RNA modifications have been reported to have a regulatory role in 7SK snRNP assembly. Pseudouridylation of 7SK snRNA on its third stem-loop at U250 was observed to be essential for stabilizing 7SK snRNP assembly as point mutation of U250 or shRNA depletion of the catalytic subunit of the pseudouridylating enzyme resulted in 7SK snRNP disruption, exchange of P-TEFb into transcriptionally active complexes, and Tat-dependent reactivation of latent HIV-1 [382]. More recently, an RNA methylation-dependent switch on 7SK conferred by the RNA methyltransferase METTL3 in response to epidermal grown factor signaling in HeLa cells was found to induce a remodeling of 7SK snRNP to release HEXIM1 and P-TEFb while facilitating the assembly of 7SK snRNA with heterogeneous nuclear ribonucleoproteins [383]. Also affirming this hypothesis are studies demonstrating that several Ser/Thr phosphatases, including PP2B, PP1α, and PPM1G, can catalyze the transient dephosphorylation of the activation loop of CDK9 at Thr186, leading to the destabilization and release of P-TEFb from 7SK snRNP [284, 378]. Dephosphorylation of pThr186 CDK9 has been proposed to be especially important for delivering P-TEFb to proviral HIV-1 [284, 384]. However, this mechanism of 7SK snRNP dissociation requires that the released P-TEFb be rapidly rephosphorylated since pThr186 CDK9 is critical for stabilizing CDK9:CycT1 heterodimerization without which CDK9 would be catalytically inactive [261, 271, 371].

PKC-θ-dependent phosphorylation of HEXIM1 at Ser158 (pSer158 HEXIM1), situated right in the middle of its bipartite RNA binding motif, is thought to occur on 7SK-free HEXIM1 and has been identified as one of the modifications that could be functionally important for preventing 7SK snRNP reassembly in T cells [379]. Biochemical experiments conducted in Jurkat T cells and employing a dominant negative PKC-θ mutant demonstrated that the activation of PKC-θ by phorbol ester, or through the TCR, can also mediate the dissociation of 7SK snRNP, albeit through mechanisms that are yet to be defined [379]. PKC-θ-dependent dissociation of 7SK snRNP and pSer158 HEXIM1 are, in turn, associated with the stimulation of P-TEFb elongation activity [379]. In a mass spectrometry-based proteomics study designed to identify posttranslational modifications that regulate 7SK snRNP assembly, we identified two adjacent tyrosine sites of HEXIM1 (Tyr271 and Tyr274) that are subject to alternative phosphorylation and whose phosphomimetic mutations are associated with the dissociation of P-TEFb and HEXIM1 from 7SK snRNA [385]. While the stable ectopic expression of the phosphomimetic HEXIM1 mutant Y271E/Y274E did not elicit a spontaneous reactivation of proviral gene expression in a Jurkat latency model, the Y271F/Y274F mutant suppressed signal-dependent proviral reactivation. Also, Y271F/Y274F HEXIM1 imparted a severe growth defect on these Jurkat T cells once they were switched to a culture medium containing low serum [385]. Although we are yet to identify the nuclear tyrosine kinase involved, these data indicate that Y271F/Y274F may block the exchange of active P-TEFb from the 7SK complex, thereby limiting the level of P-TEFb below the threshold required to support transcription elongation of the HIV-1 provirus and cellular genes. Interestingly, these tyrosine sites are situated in an unstructured region immediately upstream of the HEXIM1 C-terminal coiled-coil domain reported to form inhibitory interactions with the catalytic site of CDK9 [269, 270]. Therefore, phosphorylation of Tyr271 and Tyr274 may contribute to reversing the inhibition of CDK9 kinase. In this respect, the phosphomimetic Y271E/Y274E mutant not only abrogates 7SK snRNP assembly but also causes the exclusion of CDK9 from the nucleus, as observed in microscopic imaging experiments [385]. Thus, Tyr271/Tyr274 may mediate HEXIM1 interactions with P-TEFb, essential for reversibly repressing the enzyme’s kinase activity. Sequestration of P-TEFb within 7SK snRNP may also stabilize the retention of P-TEFb in the nucleus, allowing for its convenient exchange onto transcriptionally active genes.

### Latency reversal using TCR pathway activators

An ideal latency reversal agent (LRA) is expected to boost the transcriptional reactivation of latent HIV-1 in vivo without the unwanted and potentially harmful global T-cell activation responses elicited by TCR signaling. The few LRAs that have shown promise in reactivating latent HIV-1 in primary cell or animal models act by either targeting select TCR pathways [21, 22, 167, 240, 386, 387] or through heat shock protein stress response pathways that culminate in the nuclear mobilization of HSF1 [323, 328, 388, 389]. Similarly, LRAs that target the non-canonical NF-κB pathway and those that mimic the second messenger diacylglycerol (DAG) are not only able to reactivate latent HIV-1 but also help to reduce HIV-1 reservoir size in preclinical animal models [386, 390, 391].

We have recently reported that DAG-mimicking PKC agonists such as ingenol-3-angelate and prostratin can stimulate the biogenesis of P-TEFb to reactivate latent HIV-1 in primary CD4^+^ T cells primarily via the RasGRP1-Ras-Raf-MEK-ERK1/2 MAPK pathway rather than through PKC enzymes [240]. In the context of TCR signaling, we also showed that the ERK1/2 MAPK pathway is complemented by the PI3K-mTORC2-AKT-mTORC1 pathway in mediating TCR-induced P-TEFb expression in primary T cells (Fig. 6). By contrast, both PKC and the c-Jun MAPK pathway were found to be dispensable for generating P-TEFb or even reactivating latent HIV-1 in an ex vivo primary cell model [240].

Since the activation of PKC-θ and c-Jun MAPK can trigger NF-κB- or NFAT/AP-1-dependent T-cell activation signals that are associated with undesirable pro-inflammatory responses [354, 357], selectively targeting the RasGRP1 C1 domain might be an attractive strategy for reversing HIV-1 latency in T cells. Despite the similar overall structure of C1 domains between PKC enzymes and RasGRP proteins, subtle differences can confer differential binding and membrane association properties allowing the identification of DAG analogs that display binding selectivity. There has been a significant effort to develop synthetic DAG analogs that distinguish between various PKC enzymes or are more selective in binding RasGRP proteins [392–397]. Although synthetic DAG mimics such as bryostatin 1 analogs (bryologs), prostratin analogs, and DAG lactones have demonstrated promise as potential latency-reversing agents in cell line and primary cell models [398–402], these agents have been designed to target the C1 domains of PKC with high affinity and it is therefore likely that they their effects are elicited primarily through canonical NF-κB signaling. Identifying synthetic RasGRP1-selective C1 domain agonists able to effectively stimulate P-TEFb biogenesis in primary T cells is a promising latency reversal strategy that might circumvent undesirable cellular and systemic inflammatory responses.

## Concluding remarks

As described in this review, HIV-1 latency is inextricably linked to the cell biology of the host T cell. The main driver for entry into latency is the transitioning of infected permissive effector cells into quiescent memory cells. This transition is associated with a massive shutdown of the transcription apparatus and cellular metabolism. Consequently, reactivation of HIV-1 requires the overcoming of multiple blocks, including the reversal of epigenetic silencing factors and the mobilization of transcriptional activators. Central to this mechanism is the biogenesis of P-TEFb, the critical co-factor for Tat, which permits efficient promoter clearance and subsequent elongation.

Although HIV-1 transcription has been studied for over 30 years, this review has highlighted several poorly understood mechanisms that still need investigation. First, an improved understanding of the structural basis for promoter-proximal pausing, which may be due to a combination of structural and kinetic barriers, will lead to a better understanding of how the provirus can toggle between latency and active transcription in a suboptimally activated T-cell. Second, the detailed mechanism of P-TEFb release from the 7SK snRNP complex and its delivery to genes remains mysterious. Although the textbook models for transcription show unimolecular interactions, in fact transcription occurs in the context of the nuclear structure. Cell imaging and biochemical experiments now offer exciting opportunities to determine whether HIV-1 proviruses are recruited into transcription factories where activators of transcription initiation and P-TEFb accumulate. Finally, the central paradox of HIV transcription remains largely unsolved: how are the initial rounds of HIV-1 transcription achieved in the absence of Tat? It seems likely that the mechanism involves the recruitment of P-TEFb, but the specific mediators for P-TEFb delivery are still under investigation. Understanding how HIV-1 transcription is ignited when T cells are activated may be the key to understanding how to efficiently reverse latency.

One of the main schemes to eliminate the residual reservoir is to deliberately reactivate the latent HIV-1 proviruses to enable clearance of persisting latently infected cells—the “Shock and Kill” strategy. Several recent preclinical studies in animal experimental models have generated promising findings in demonstrating a proof-of-concept for the Shock and Kill approach [386, 390, 403]. However, it has been challenging to identify key rate-limiting steps that can be targeted to efficiently reverse latency. Given the numerous steps involved in reactivating HIV-1 transcription at the molecular level, it is not surprising that genetic screens for HIV-1 latency factors have uncovered a bewildering array of mechanisms controlling HIV-1 transcription [72, 404–412]. Similarly, although a wide range of latency-reversing agents have been identified, many with distinct mechanisms of action [413, 414], LRAs have, so far, only induced transient viral production in infected individuals but have failed to lower the size of the latent reservoir [414].

Thus, despite extensive efforts, “Shock and Kill” has largely failed because efficient, non-toxic LRAs remain to be discovered. A key message from this review is that it seems unlikely that efficient viral reactivation can be achieved without activating both HIV-1 transcriptional initiation and elongation. Thus, carefully designed combinations of LRAs will probably be required to achieve viral reactivation. Several directed screens for synergies between latency-reversing agents have recently been completed and have uncovered promising combinations of small molecules and biologics [21, 410, 415]. Alternatively, selective activation of T-cell signaling pathways, such as non-canonical NF-κB, MAPK ERK, and mTORC1, may offer an efficient way to reverse HIV latency while minimizing toxicities.

## Data Availability

Not applicable.

## Abbreviations

7SK snRNP: 7SK small nuclear ribonucleoprotein complex
AKT: Protein kinase B
AP1: Activator protein-1
ART: Antiretroviral therapy
ATACseq: Assay for transposase-accessible chromatin with sequencing
ATI: Analytical treatment interruption
BAF: BRG-/BRM-associated factor
BRD4: Bromodomain-containing protein 4
CAF-1: Chromatin assembly factor 1
CBP: CREB-binding protein
CBX4: Chromo box 4
CDK7: Cyclin-dependent kinase 7
CDK9: Cyclin-dependent kinase 9
CTD: C-terminal domain
CycT1: Cyclin T1
DAG: Diacylglycerol
DNMT1: DNA methyltransferase 1
DNMT3a: DNA methyltransferase 3a
DSIF: DRB sensitivity-inducing factor
EDITS: Envelope detection of in vitro transcription sequencing
EHMT2: Euchromatic histone lysine methyltransferase 2
EMT: Effector-to-memory transition
ERK: Extracellular signal-regulated kinase
EZH2: Enhancer of zeste 2
FACT: Facilitates chromatin transcription complex
HIV-1: Human immunodeficiency virus
H2Bub1: Histone H2B mono-ubiquitination
H3K4me3: Histone H3 Lys4 tri-methylation
H3K27Ac: Histone H3 Lys27 acetylation
H3K27me2/3: Histone H3 Lys27 di- or tri-methylation
H3K79me2/me3: Histone H3 Lys79 di- or tri-methylation
H3pS10: Histone H3 Ser10 phosphorylation
H4K20me1: Histone H4 Lys20 mono-methylation
HDAC: Histone deacetylase
HEXIM1/2: Hexamethylene bis-acetamide inducible protein 1/2
HSF1: Heat shock factor protein 1
IPDA: Intact proviral DNA assay
JAK: Janus kinase
JNK: C-Jun N-terminal kinase
KAT5: Lysine acetyltransferase 5
LARP7: La-related protein 7
LRA: Latency-reversing agent
LTR: Long terminal repeat
MAPK: Mitogen-activated protein kinase
MBD2: Methyl-CpG binding domain protein 2
MEPCE: Methylphosphate capping enzyme
MHC: Major histocompatibility complex
mTOR: Mammalian target of rapamycin
mTORC1: Mammalian target of rapamycin complex 1
mTORC2: Mammalian target of rapamycin complex 2
NELF: Negative elongation factor
NFAT: Nuclear factor of activated T cells
NF-κB: Nuclear factor kappa B
NMR: Nuclear magnetic resonance
Nuc-0, 1 and 2: Nucleosome-0, -1 and -2
NuRD: Nucleosome remodeling and deacetylase complex
p300: Histone acetyltransferase p300
PAF1c: Polymerase-associated factor 1 complex
PBAF: Polybromo-associated BAF (BRG-/BRM-associated factor)
PBMCs: Peripheral blood mononuclear cells
PDK1: Phosphoinositide-dependent kinase 1
PH domain: Pleckstrin homology domain
PI3K: Phosphoinositide-3-kinase
PKC: Protein kinase C
PLC-γ: Phospholipase C gamma
PIP_2_: Phosphatidylinositol-4,5-bisphosphate
PIP_3_: Phosphatidylinositol-3,4,5-triphosphate
PML: Promyelocytic leukemia
PRC1: Polycomb repressive complex 1
PRC2: Polycomb repressive complex 2
P-TEFb: Positive transcription elongation factor b
PTM: Post-translational modification
Q4PCR: Quadruplex quantitative PCR
Q-VOA: Quantitative viral outgrowth assay
RasGRP: Ras guanine nucleotide-releasing protein
RBF-2: Ras-responsive binding factor 2
RNA FISH: RNA fluorescence in situ hybridization
RNAP II: RNA polymerase II
RNF20/40: E3 ligases RING finger proteins 20 and 40
SEC: Super elongation complex
SIN1: Stress-activated MAP kinase interacting protein 1
SMYD2: SET and MYND domain-containing protein 2
SOS: Son of sevenless
Sp1: Specificity protein 1
SPT6: Suppressor of Ty 6 homolog
STAT5: Signal inducer and activator of transcription 5
SWI/SNF: SWItch/sucrose non-fermentable
TAR: Trans-activation response element
TCR: T-cell receptor
TFIIH: Transcription factor II H
TILDA: Tat/Rev-induced limited dilution assay
UBE2O: Ubiquitin-conjugating enzyme E2 O
UHRF1: Ubiquitin-like with PHD and RING finger domains 1
UTX/KMDA: Histone H3 trimethyl Lys27 demethylase
